# Cell shape and the microenvironment regulate nuclear translocation of NF-κB in breast epithelial and tumor cells

**DOI:** 10.15252/msb.20145644

**Published:** 2015-03-03

**Authors:** Julia E Sero, Heba Zuhair Sailem, Rico Chandra Ardy, Hannah Almuttaqi, Tongli Zhang, Chris Bakal

**Affiliations:** 1 Chester Beatty Laboratories, Division of Cancer Biology, Institute of Cancer ResearchLondon, UK; 2 Oxford Centre for Integrative Systems Biology, Department of Biochemistry, University of OxfordOxford, UK

**Keywords:** Bayesian, breast cancer, morphology, NF-κB, RhoA

## Abstract

Although a great deal is known about the signaling events that promote nuclear translocation of NF-κB, how cellular biophysics and the microenvironment might regulate the dynamics of this pathway is poorly understood. In this study, we used high-content image analysis and Bayesian network modeling to ask whether cell shape and context features influence NF-κB activation using the inherent variability present in unperturbed populations of breast tumor and non-tumor cell lines. Cell–cell contact, cell and nuclear area, and protrusiveness all contributed to variability in NF-κB localization in the absence and presence of TNFα. Higher levels of nuclear NF-κB were associated with mesenchymal-like versus epithelial-like morphologies, and RhoA-ROCK-myosin II signaling was critical for mediating shape-based differences in NF-κB localization and oscillations. Thus, mechanical factors such as cell shape and the microenvironment can influence NF-κB signaling and may in part explain how different phenotypic outcomes can arise from the same chemical cues.

## Introduction

Cell morphology reflects the balance of internal and external chemical and physical cues, which in turn provide the context for signaling events. The shape of a cell can regulate differentiation, proliferation, survival, and motility (Chen *et al*, 1997; McBeath *et al*, 2004; Discher *et al*, 2009; Sero *et al*, 2011), and the role of morphology in tumor progression has become apparent in recent years. For example, cell shape is greatly influenced by the stiffness of the extracellular matrix (ECM), which correlates with metastasis and prognosis in breast cancer (Kass *et al*, 2007; Mouw *et al*, 2014). Indeed, the morphology of cells has long been the primary method of diagnosing and staging tumors in the clinic. While mechanosensitive transcriptional regulators, such as YAP, have been shown to link shape information with gene expression (Cordenonsi *et al*, 2011; Dupont *et al*, 2011; Aragona *et al*, 2013), the mechanisms by which cellular fates are determined by cell shape are still poorly understood.

Nuclear factor kappa B (NF-κB) is a key transcription factor involved in mediating inflammation, cellular stress responses, and tumor progression. NF-κB also controls cell and tissue morphogenesis, including proliferation and branching of the mammary gland (Brantley *et al*, 2001). Chronic expression of the inflammatory cytokine tumor necrosis factor-alpha (TNFα), a potent NF-κB activator (Van Antwerp *et al*, 1996), can drive cancer metastasis by inducing epithelial-to-mesenchymal transition (EMT) and tumor cell migration (Wu & Zhou, 2010). NF-κB dimers are sequestered in the cytoplasm by inhibitory I-κB until activated by cellular stress or cytokines (Gilmore, 2006). Binding of TNFα to cell surface receptors leads to recruitment of adaptors (TRAFs and RIP) and activation of IKK kinases, which phosphorylate I-κB and target it for proteolytic degradation. Once released from inhibition, NF-κB can translocate to the nucleus, bind DNA, and induce the expression of target genes, including I-κB.

Following TNFα stimulation, the nuclear to cytoplasmic ratio of NF-κB has been shown to exhibit damped oscillations (Ashall *et al*, 2009; Kalita *et al*, 2011; Zambrano *et al*, 2014). A number of studies have demonstrated that the dynamics of these oscillations determine its physiological effects. For example, target gene expression depends on the persistence of NF-κB oscillations (Sung *et al*, 2009; Tay *et al*, 2010), and NF-κB dynamics have been reported to encode gene-specific transcription patterns (Lee *et al*, 2014). Notably, however, recent studies appear to suggest these oscillations may be cell type or context specific, suggesting that biophysical factors can impact NF-κB dynamics (Kearns *et al*, 2006). Moreover, NF-κB activation and dynamics are heterogeneous on the single cell level, even in isogenic populations (Nelson *et al*, 2004). This heterogeneity may be due to stochastic noise and/or may be regulated deterministically.

Mechanical forces can activate NF-κB in many cell types, including muscle, lung, and vasculature (Chen *et al*, 2003; Copland & Post, 2007), and this signaling pathway is sensitive to perturbation of F-actin and microtubules (MT), the main components of the cytoskeleton (Rosette & Karin, 1995; Nemeth *et al*, 2004). While these findings indicate that NF-κB activity is affected by cellular architecture, the exact mechanisms that underpin its mechanosensitivity are unclear. In this study, we asked whether cell shape and context can influence NF-κB and whether differences in shape can explain any of the cell-to-cell variability observed in its activation. We used high-content analysis (HCA) to quantitatively measure cell morphology and transcription factor (TF) localization in hundreds of thousands of single cells per experiment in cell lines derived from human breast tumor and non-tumor tissues. Using this compendium of data, we built statistical Bayesian network models that exploit intrinsic heterogeneity present in cellular populations to uncover dependencies between cell shape, context, and TF intensity features. We then tested predictions from network models using chemical, physical, and genetic methods. This study reveals that cell shape and microenvironmental factors are determinants of NF-κB translocation dynamics that contribute to cell-to-cell heterogeneity. We propose that cell shape-mediated regulation of NF-κB plays a key role in breast epithelial development and tumor progression.

## Results

### Experimental settings and image analysis

**Figure 1:**
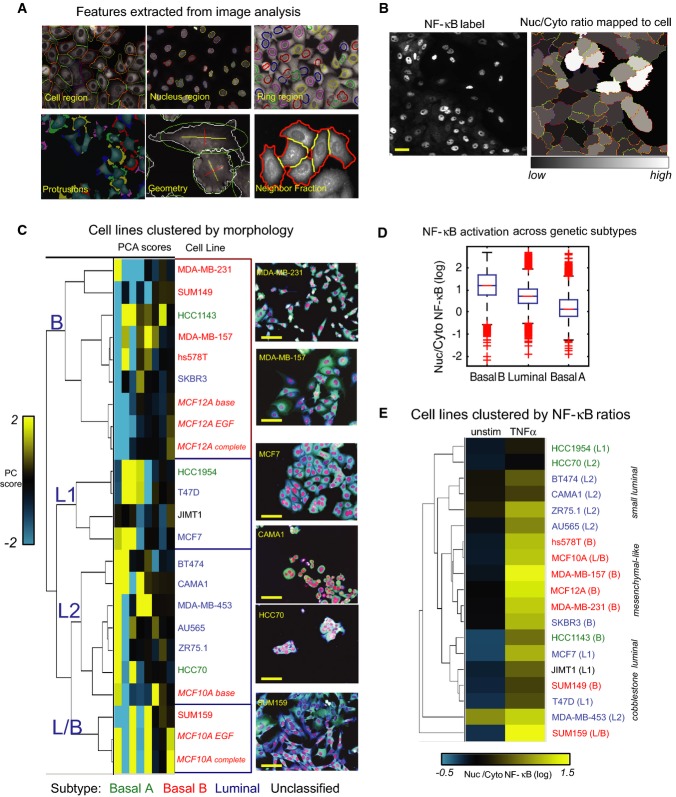
Characterization of cell morphology and NF-κB activation in breast cell lines

We used HCA to quantitatively profile the morphology and NF-κB nuclear localization (which serves as a proxy for its activation), in 17 breast cancer and two non-tumor cell lines (Table1). Nuclei and cell bodies were stained with fluorescent labels and imaged by confocal microscopy (see Materials and Methods). Cellular regions (nucleus, cytoplasm, membrane, perinuclear ring) were automatically segmented and measured (Fig1A, top). All cells were grown in ‘base’ medium, and the non-tumor lines MCF10A and MCF12A were also cultured ‘complete’ medium, as MCF10A cells required EGF to proliferate (see Materials and Methods). This dataset contained 307,643 cells, and a total of 77 shape and context features were measured for each cell (Fig1A). These include geometric features such as area, roundness, length/width; measurement of protrusions and ‘ruffliness’, which detects variations in membrane intensity; measures of cell polarity, such as the distance between the centroids of the nucleus and the cell body (centers distance); measures of context including colony size and neighbor fraction (NF), the proportion of a cell's boundary that is in contact with other cells; and fluorescence intensity in each subcellular region. On the third day in culture, cells were treated with or without TNFα for 1 h or 5 h to capture the first peak and later steady state of NF-κB activation (see Fig4E). NF-κB activation state can be inferred by the ratio of nuclear to cytoplasmic (perinuclear, i.e., ring region) p65/RelA fluorescence intensity, which we hereafter refer to as the ‘NF-κB ratio’ (Nelson *et al*, 2004) (Fig1B).

**Table 1:** Breast tumor and non-tumor cell lines.

Cell line	Genetic subtype	ER	PR	Her2	TP53	N-cadherin	Shape cluster
hs578T	BaB	−	[−]	−	+M	++	B
MCF10A (complete medium)	BaB	−	[−]	−	+/−WT	++	L/B
MCF10A (base medium)	BaB	−	[−]	−	+/−WT	++	L2
MDAMB157	BaB	−	[−]	−	−	++	B
MDAMB231	BaB	−	[−]	−	++M	−	B
SUM149	BaB	[−]	[−]	−	[+]	+	B
SUM159	BaB	[−]	[−]	−	[−]	++	L/B
HCC1143	BaA	−	[−]	−	++M	++	B
HCC1954	BaA	−	[−]	+	[+/−]	−	L1
HCC70	BaA	−	[−]	−	++M	−	L2
AU565	Lu	−	[−]	+	+WT	−	L2
BT474	Lu	+	−	+	+	−	L2
CAMA1	Lu	+	[−]	−	+	−	L2
MCF7	Lu	+	[+]	−	+/−WT	−	L1
MDAMB453	Lu	−	[−]	−	−WT	−	L2
SKBR3	Lu	−	[−]	+	+	−	B
T47D	Lu	+	[+]	−	++M	−	L1
ZR75.1	Lu	+	[−]	−	−	−	L2
JIMT1		−	[−]	+	++	−	L1

Gene cluster: Lu = luminal, BaA = Basal A, BaB = Basal B. ER/PR/HER2/TP53 status: +/− from protein and mRNA expression; [−] inferred from mRNA expression; M = mutant, WT = wild-type (Neve *et al*, 2006). N-cadherin status: +/− from mRNA expression and immunofluorescence (Supplementary Fig S1B). Shape clusters assigned from hierarchical clustering shown in Fig1D.

Cell segmentation and shape features.

NF-κB staining in MCF10A cells (left) and nuclear/cytoplasmic ratios mapped as gray-scale pixel values to segmented cells (right). Scale bar = 20 μm.

Hierarchical clustering of the first 8 principal component scores for each cell line based on geometric and context features. Genetic subtype is indicated by color. Representative images (20×) of cells from each cluster are shown at the right (red: DAPI, green: NF-κB, blue: DHE). Scale bar = 30 μm.

NF-κB ratios (TNFα 1 h) in cell lines of different genetic subtypes (box and whisker plot). 14 replicates wells per cell line. Basal A: 3 lines, Basal B: 7 lines, Luminal: 8 lines.

Cell lines clustered by nuclear/cytoplasmic NF-κB ratio in the presence or absence of TNFα.

### Characterization of cell lines by morphology

The cell lines profiled represent a variety of phenotypes. While some can be easily designated epithelial-like or mesenchymal-like, others have intermediate phenotypes or consist of a mixture of shapes. Principal component analysis (PCA) was performed on well averages of geometric, protrusion, polarity, and context features to reduce the dimensionality of the data. The average scores of the first eight PCs for each cell line, including MCF10A and MCF12A cells in different media, were grouped by hierarchical clustering (centered correlation, centroid linkage) (Fig1C). Replicate wells also clustered together, showing good reproducibility of feature measurements (Supplementary Fig S1C).

Two major groups emerged from this clustering. The first major cluster (B) contained mostly myoepithelial Basal B cell lines, and the second (L) contained mostly luminal lines (Neve *et al*, 2006) (Fig1C). The Basal B group was characterized by significantly larger cell area (2,269 ± 672 μm^2^ [mean ± SD]), low nuclear/cytoplasmic area (A_nuc_/A_cyto_; 0.15 ± 0.029), low NF (0.36 ± 0.1), and high ruffliness (0.215 ± 0.023) compared with the luminal lines (*P *<* *0.01) (Supplementary Fig S1). The luminal cluster contained three sub-groups with epithelial-like morphologies. The first sub-group (L1) contained lines with classic ‘cobblestone’ epithelial morphology, such as MCF7, that grew in colonies with extensive cell–cell contacts (NF = 0.56 ± 0.038) and were significantly larger in area (1,788 ± 308 μm^2^) than the L2 group (Fig1D and Supplementary Fig S1G). The L2 sub-group contained smaller cells (835 ± 173 μm^2^) that grew in colonies (e.g. HCC70) or clusters (e.g. CAMA1) (NF = 0.46 ± 0.087). Non-proliferative MCF10A cells (base medium) were also in this group. The final sub-group (L/B) contained two Basal B lines with luminal morphologies, SUM159, and proliferative MCF10A cells. This group may represent an intermediate or progenitor phenotype, as these lines are derived from the myoepithelial layer and contain stem cell-like subpopulations (Fillmore & Kuperwasser, 2008).

Membership in a morphological cluster was not correlated with ER, PR, HER2, or TP53 status. However, clusters did correspond to expression of N-cadherin, a marker of EMT and poor prognosis in breast cancer (Andrews *et al*, 2012) (Table1 and Supplementary Fig S1B). None of the luminal cell lines were positive for N-cadherin, whereas both cell lines of the L/B group and most of the lines in the B group were. Furthermore, the only Basal A line in the B morphological cluster, HCC1143, was N-cadherin positive. Morphological profiling was therefore not only able to recapitulate molecular subtypes determined by gene expression profiles (basal/luminal), but also discriminated between myoepithelial-derived basal cell lines that were positive or negative for an important EMT marker and pointed to a further distinction between Luminal cell lines.

### NF-κB activation in response to TNFα scales with subtype and cell shape

Ranking cell lines by NF-κB ratio shows that Basal B lines stimulated with TNFα for 1 h (roughly the first peak of NF-κB activation) had predominantly nuclear staining (nuclear/perinuclear ratio > 0.8), compared with luminal (0.2–0.8) and Basal A lines (0.1–0.6). One-way ANOVA with a multi-comparison procedure confirmed significant differences in NF-κB localization between genetic subtypes (*P *<* *0.01) (Fig1D). Importantly, we also observed a relationship between cell morphology and NF-κB response within genetic subtypes. Luminal cell lines with cobblestone morphology (L1) had significantly lower unstimulated NF-κB ratios and greater fold change after TNFα stimulation than cell lines in the L2 sub-group (*P *<* *0.01) (Fig1E). Clustering cell lines by stimulated and unstimulated NF-κB ratios shows the distinction between L1 and L2 sub-groups, as these lines did not cluster together (Fig1E). Furthermore, NF-κB activation in the Basal A cell lines followed the morphological trend: the B group HCC1143 cells had the highest NF-κB ratio after TNFα, followed by the L1 line HCC1954, and finally the L2 line HCC70 (Fig1E and Supplementary Fig S2A).

### Relationship between NF-κB and morphology in single cells

**Figure 2:**
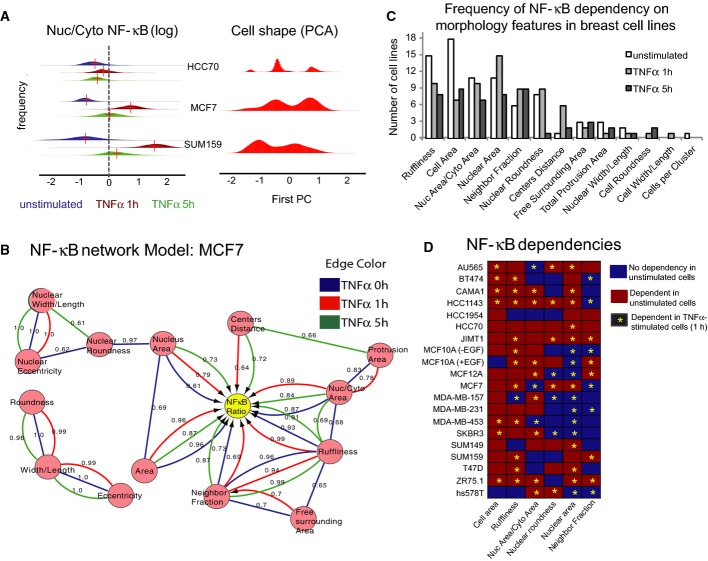
Bayesian network dependency models infer connections between shape and transcription factor localization using intrinsic heterogeneity of single cells

Given the differences in NF-κB activation between differently shaped breast cell lines, we next set out to determine whether NF-κB activation was related to morphology on the level of single cells. The distribution of NF-κB ratios varied between cell lines and conditions (Fig2A (left) and Supplementary Fig S2A), and in some cases, the cell-to-cell differences ranged over orders of magnitude (log ratio −1 to > 1). Moreover, all cell lines were morphologically heterogeneous. Figure2A (right) and Supplementary Fig S2B show the distribution of first principal component (PC1) scores of single cells based on 77 geometric shape and context features. Cell shape distributions were multi-modal, suggesting the existence of a finite number of morphological states (Yin *et al*, 2013; Sailem *et al*, 2014), and each cell line showed a different degree of heterogeneity.

Left: Distribution of nuclear/cytoplasmic NF-κB ratios in single cells ± TNFα (blue = unstimulated, red = TNFα 1 h, green = TNFα 5 h) showing distribution of values for each cell line. Right: Distribution of first principal component (PC) of morphology features showing multi-modal distribution of cell shapes.

Example Bayesian network (MCF7). Edge (line/arrow) color denotes treatment conditions. Arrows indicate direction of dependency, and lines indicate interactions where direction cannot be determined. Numbers denote confidence (see Materials and Methods).

Frequency of NF-κB ratio dependencies by cell line for the most commonly connected features ± TNFα (1 h).

NF-κB dependencies on morphological factors detected in each condition. Red = dependency detected in unstimulated condition. Asterisk = dependency detected in TNFα-stimulated cell (1 h).

We first asked whether any shape and context features differed significantly between cells with high and low NF-κB ratios in each cell line. Interestingly, many features varied consistently in cell lines (Supplementary Fig S2C and Supplementary Materials and Methods). Unstimulated cells with nuclear NF-κB (high ratio) tended to have significantly higher ruffliness, lower nuclear roundness, smaller nuclear and cell areas, and fewer neighboring cells than those with low NF-κB ratios in many cell lines (Supplementary Fig S2C). However, linear correlations between NF-κB ratios and morphological features were poor (Supplementary Fig S2D), which suggests that the relationship between NF-κB and cell morphology is complex and non-linear.

### Bayesian network models

To investigate the relationship between cell shape, microenvironmental factors, and NF-κB, we used Bayesian network modeling to identify dependencies between variables. Bayesian networks are a class of graphical models that can capture non-linear relationships between interacting components using conditional probabilities (Friedman *et al*, 2000; Sachs *et al*, 2005). Network models were built for each cell line ± TNFα using the 17 significant features that were most frequently significantly different between cells with high and low NF-κB ratios across cell lines, resulting in 60 network models (Supplementary Fig S2C). An example network model (MCF7) is shown in Fig2B, where each feature is represented as a node and interactions by lines or arrows. An arrow pointing from A to B indicates that B is dependent upon A; that is, changes in A affect the value of B. Numbers denote the confidence of the interaction (see Supplementary Materials and Methods). Undirected arrows indicate an interaction where the dependency cannot be determined. In MCF7 cells (Luminal genetic subtype, L1 morphological group), NF-κB ratio was dependent on cell area, nucleus area, ruffliness, A_nuc_/A_cyto_, and NF in unstimulated cells, as well as on centers distance (distance between cell and nuclear centroids) after TNFα stimulation (confidence > 0.6).

The statistical dependency of NF-κB localization on shape features varied between cell lines and conditions (Fig2C and D), but some dependencies were more frequent than others. The most commonly connected features in unstimulated cell lines were cell area (18 cell lines), ruffliness (15), nuclear area (11), A_nuc_/A_cyto_ (11), and nuclear roundness (8 lines) (Fig2C). In TNFα-stimulated cells, the most common dependencies were nuclear area (15), A_nuc_/A_cyto_ (10), ruffliness (10), NF (9), and nuclear roundness (9). In some cell lines, NF-κB ratio was more dependent on morphological factors before stimulation (Basal A lines HCC70 and HCC1954), while in other lines it was more dependent on shape after addition of TNFα (Basal B lines MDA-MB-157 and hs578T). TNFα stimulation reduced the frequency of dependency on cell area and ruffliness, but increased dependency on nuclear morphology and NF. These findings indicate that the effects of cell morphology on NF-κB localization are largely conserved across cell lines but can vary depending on whether cells are responding to cytokine stimulation (± TNFα).

### YAP, but not Jun, activation is dependent on cell morphology

**Figure 3:**
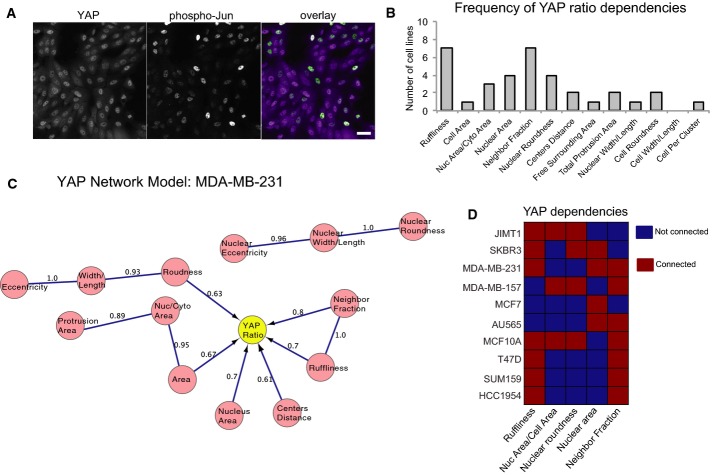
The statistical dependency of YAP on cell shape

To test the validity of this approach, we asked whether Bayesian network models could detect connections between cell shape features and localization of YAP, another transcription factor that is activated by nuclear translocation and is known to be regulated by cell density, spreading, and mechanical force (Fig3A, left) (Halder *et al*, 2012). Nuclear/cytoplasmic YAP ratios varied widely across ten cell lines (Fig3B) and were not significantly affected by 1-h TNFα treatment (Supplementary Fig S3A). YAP ratio was dependent on many of the same shape and context features as NF-κB ratio, specifically NF, ruffliness, and nuclear morphology features (7/10 lines each), but the networks were not identical. In MDA-MB-231 cells (genetic subtype Basal B, morphological group B), for example, YAP ratio was dependent on cell roundness, whereas NF-κB ratio was not (Figs2D and 3C). In MCF7 cells (Luminal, L1), NF-κB ratio was also highly connected to shape, but YAP ratio was only dependent on nuclear area (Figs2 and 3D). YAP ratio was most often dependent on NF and ruffliness. These data demonstrate that our experimental and analytical approach captures the known relationships between YAP nuclear localization and cell shape. Moreover, we find that the amount of nuclear YAP and NF-κB is dependent on overlapping, but not identical, aspects of cell morphology.

MCF10A cells stimulated with TNFα labeled for YAP and phospho-Jun. Scale bar = 20 μm.

Frequency of YAP ratio dependencies by cell line for the most commonly connected features.

Example Bayesian network model for YAP ratio (MDA-MB-231 cells).

Dependencies of YAP ratio on the features that are most frequently connected to YAP ratio in unstimulated conditions. Red = dependency detected.

In order to determine whether the activity of other branches of the TNFα signaling pathway are dependent on cell shape, we measured levels of phosphorylated Jun, a transcription factor that is activated downstream of TNFR ligand binding by phosphorylation of Jun N-terminal kinase JNK (Supplementary Fig S3B). While phosphorylated Jun accumulated in cell nuclei following TNFα stimulation, Bayesian models did not infer dependency between nuclear pJun intensity and any morphological features in any of the ten cell lines tested (Supplementary Fig S3C). Furthermore, nuclear/cytoplasmic pJun and NF-κB ratios were not correlated in TNFα-stimulated MCF10A cells expressing GFP-p65/RelA (*R*^2^ < 0.05, n = 594 GFP-positive cells) (Supplementary Fig S3D). (In comparison, the *R*^2^ for NF-κB and YAP ratios was typically 0.3 in unstimulated cells and 0.5 in TNFα-stimulated MCF10A cells.) These data indicate that although NF-κB translocation downstream of TNFα is shape sensitive, Jun phosphorylation downstream of TNFR is not.

Importantly, the finding that Jun activation is not dependent on cell shape or microenvironment also suggests that the statistical correlations between shape and NF-κB activation are not simply due to the fact that some cells express more TNFR than others. This notion is supported by the fact we found no strong correlations (*R*^2^ > 0.5) between NF-κB ratios and mRNA levels of TNFR, or other members of the NF-κB signaling pathway (I-κB, RelA, RelB, NFKB1, NFKB2, TRAF2, TRAF5, TNIK, or four IKK genes [α, β, γ, ε]) in any treatment condition (unstimulated, 1 h, or 5 h TNFα) using microarray data generated from 18 of the 19 cell lines used in this study (Supplementary Table S1) (Grigoriadis *et al*, 2012) (no data were available for AU565 cells, which were obtained from ATCC). These results suggest that the differences in NF-κB localization between and within cell lines are not simply due to cells’ ability to detect TNFα.

### Drug-induced cytoskeleton modification affects cell shape and NF-κB

**Figure 4:**
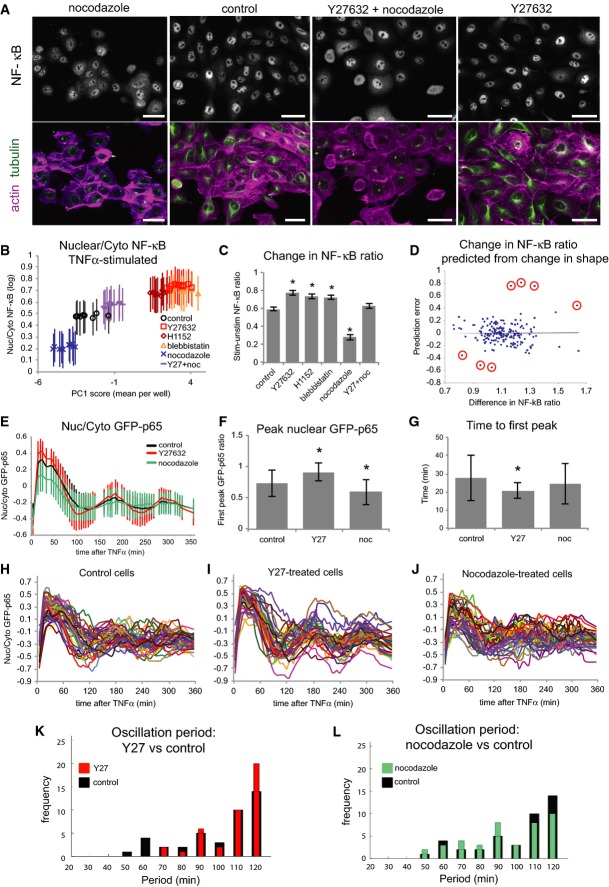
Cytoskeleton-modifying drugs affect NF-κB activation and dynamics

We next asked whether inducing cell shape changes by chemically altering the cytoskeleton would affect NF-κB. Non-tumor MCF10A cells were treated with Y-27632 (Y27), H1152, blebbistatin (Blebb), and/or low doses of nocodazole (Noc) prior to TNFα stimulation. Y27 and H1152 induce F-actin depolymerization by inhibiting the activity of ROCK, a RhoA effector, and Blebb disrupts F-actin contractility by blocking myosin II ATPase (Kovacs *et al*, 2004). Noc inhibits MT polymerization and can induce activation of RhoA via GEF-H1 (Chang *et al*, 2008). Y27, H1152, and Blebb treatment induced cell spreading and disassembly of cell–cell adhesions, whereas Noc induced rounding up and an increase in E-cadherin staining at cell–cell contacts (Fig4A and Supplementary Fig S4D). The first PC scores of shape and context features (well averages) illustrate the opposite effects of Y27/H1152/Blebb and Noc on cell morphology (Fig4B). These drugs also had contrasting effects on NF-κB. Y27, H1152, and Blebb increased, whereas Noc decreased, NF-κB nuclear localization after TNFα stimulation (Fig4B and C, Supplementary Fig S4A and B). Interestingly, pre-treatment with Y27 and Noc together resulted in similar cell shape to controls (by PC1 score) and ‘rescued’ NF-κB activation. Consistent with this model, stabilizing MTs with a high dose of taxol also induced cell rounding and reduced NF-κB activation (Supplementary Fig S4A–C). These data demonstrate that chemical modulation of cytoskeletal tension, and the concomitant effects on cell shape, can influence NF-κB localization in response to TNFα.

MCF10A cells ± Noc and/or Y27, in order of average PC1 scores from left to right as in (B). Scale bar = 20 μm.

Average NF-κB ratio per well by average shape (PC1) score (+ TNFα) (± SD). *n* = 840 ± 48 cells/well.

Fold change in NF-κB ratio between stimulated and unstimulated cells (± SD). *n* = 10 replicate wells/condition. *P <* *0.01 compared with control (Student's *t*-test). *n *=* *840 ± 48 cells.

Fold change of NF-κB ratios predicted from changes in three cell shape features (NF, ruffliness, A_nuc_/A_cyto_) by multivariate regression analysis in 176 cell line/drug treatment conditions (Supplementary Tables S2 and S3 and Supplementary Dataset S3). Seven cases in which changes in cell shape did not accurately predict changes in NF-κB ratio are circled.

Average nuclear/cytoplasmic GFP-p65 ratio at each time point in live cells (± SD). *n* = 40 cells/condition.

Average amplitude of first peak GFP-p65 ratio (± SD). *n* = 40 cells/condition. *P <* *0.01 (Student's *t*-test).

Average time to first peak GFP-p65 ratio (± SD). *n* = 40 cells/condition. *P <* *0.01 (Student's *t*-test).

Single cell traces of GFP-p65 ratios over time (H–J). Wavelet analysis plots show frequencies of oscillation periods in Y27-treated (red) or Noc-treated (green) cells versus control (black) (t = 300 min) (K, L).

### Prediction of changes in NF-κB from changes in morphology

To further validate that the effect of cytoskeleton-modifying drugs affected NF-κB activation through shape changes, we treated 10 cell lines (nine breast cell lines plus HeLa cells for comparison) with drugs that were expected to alter cell shape and cytoskeletal tension, including Y27, Noc, and Blebb (Supplementary Tables S2 and S3). In general, NF-κB ratios increased in cells treated with ROCK inhibitors and decreased in cells treated with nocodazole, but different lines showed different sensitivities to drugs (see Supplementary Dataset S3). We used multilinear regression with tenfold cross-validation to predict the fold change in NF-κB ratio from the fold change in morphological features (see Supplementary Materials and Methods), resulting in the following equation: 

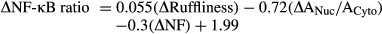
NF-κB ratio was negatively associated with A_Nuc_/A_Cyto_ and NF and positively associated with ruffliness. This model was able to predict the fold difference in average NF-κB with *R*^2^ = 0.37, low error variance (0.033), and a very significant *P* value (2.25 × 10^−17^) (Fig4D). The average error between cross-validation samples was 0.0172 (± 0.0077), and residuals were normally distributed. Changes in NF-κB were explained by changes in shape in the majority of cases. The overall goodness of fit in this statistical model strongly suggests that cell area, protrusiveness, and cell–cell contact all impact NF-κB activation. Only seven cases were not within the 95% confidence interval of the predicted value (Fig4D, circled). Three of these, in which NF-κB ratios were higher than expected based on changes cell morphology, were Y27-treated HCC1954 cells (Basal A, L1) stimulated with TNFα. The cases with lower than predicted NF-κB ratios were HCC1954, JIMT1 (unclassified, L1), and T47D (Luminal, L1) cells treated with nocodazole. HCC1954 cells had very low NF-κB activation compared with other L1 morphology group lines in the absence of ROCK inhibitor, which may indicate an inhibitory effect of RhoA signaling on NF-κB in these cells.

### Cell shape and the microenvironment regulate NF-κB translocation dynamics

To investigate how changes in cell shape affect the dynamic behavior of NF-κB, MCF10A cells were transiently transfected with GFP-p65, selected by FACS, and imaged over 6 h at 5-min intervals after addition of TNFα (Fig4E and Supplementary Movies). NF-κB ratios (nuclear/perinuclear GFP intensity) were measured for 40 cells in each condition. Y27 treatment caused an increase in nuclear NF-κB immediately after addition of TNFα, whereas Noc treatment significantly decreased the amplitude of the first peak (Fig4F). Unexpectedly, the initial wave of nuclear localization was more rapid and less variable in Y27-treated cells (Fig4G). Consistent with reports in other cell types, damped oscillations with a period of 110–120 min were observed in all conditions, with higher amplitudes in Y27-treated and lower amplitudes in Noc-treated cells (Fig4H–J) (Ashall *et al*, 2009; Zambrano *et al*, 2014). The first peak, at around 30 min, was followed by a rapid (30–40 min) then steady (60–80 min) decrease in nuclear NF-κB.

Y27 treatment resulted not only in greater amplitude but also less variability in oscillations, whereas Noc had the opposite effect. To compare NF-κB translocation dynamics in different conditions, wavelet analysis was used to estimate the instantaneous periods of oscillation at each time point (*t* = 300 min shown in Fig4K and L). Control cells showed a high frequency of 110–120 min oscillations, with smaller peaks at 60 and 90 min (Fig4K and L, black bars). Y27 increased the frequency of 120-min oscillations and reduced the frequency of shorter periods (Fig4K). In contrast, Noc treatment reduced the frequency of 110–120 min oscillations and increased the frequency of shorter periods (Fig4L). These data suggest that Noc treatment could drive NF-κB toward a non-oscillating steady state, whereas Y27 treatment could enhance the cycle of nuclear import and export. Thus, NF-κB nuclear translocation dynamics are sensitive to cell shape and the actin cytoskeleton.

### Network models predict NF-κB and YAP sensitivity to cell–cell contact

**Figure 5:**
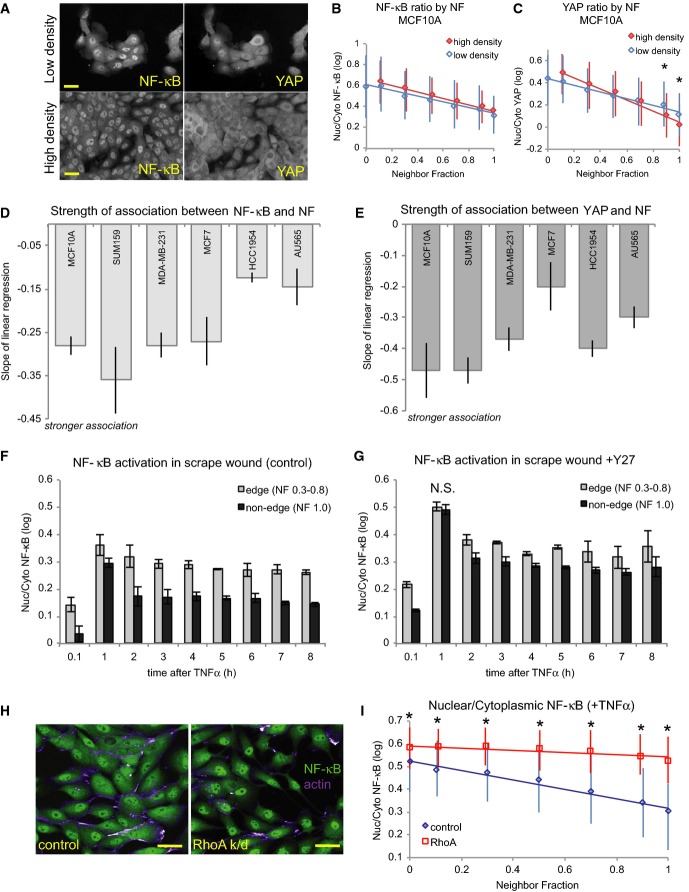
NF-κB and YAP sensitivities to cell density are consistent with predictions from network models

Although statistical modeling showed that differences in NF-κB nuclear localization could be explained by changes in shape, we cannot exclude the possibility that drugs had other effects on upstream signaling. We therefore used non-chemical methods to alter cell morphology. To test dependencies on cell–cell contact (neighbor fraction; NF), a subset of six cell lines were seeded at four different densities and stimulated with TNFα for 1 h and stained for both NF-κB and YAP (Fig5A). This gave a wider distribution of NF values and enriched populations in phenotypes that were rare in the initial screen, such as MDA-MB-231 cells with high NF and MCF7 cells with low NF (Supplementary Fig S5A and B).

A MCF10A cells plated at low and high densities. Scale bar = 20 μm.

B, C MCF10A cells from high- and low-density cultures showing average NF-κB (B) and YAP (C) ratios by NF (± SD). All wells were treated with TNFα. *P <* *0.01, difference between high- and low-density wells (Student's *t*-test).

D, E Strength of the relationships between NF-κB ratio (D) or YAP ratio (E) and neighbor fraction shown by the slopes of the best-fit regression lines between TF ratio and NF (± SD). *n* = 4 density replicates (> 1,200 cells each) per line (see Supplementary Fig S5D and E).

F, G NF-κB ratios in edge and non-edge cells of scrape-wounded monolayers of MCF10A cells stimulated with TNFα at the designated times after wounding in control (F) and Y27-treated (G) conditions. N.S. = no significant difference. Where not indicated otherwise, *P *<* *0.01 between edge and non-edge cells (Student's *t*-test).

H Mock-transfected (control) and RhoA-depleted (RhoA k/d) cells stimulated with TNFα (1 h) and stained for NF-κB (green) and F-actin (purple). Scale bar = 30 μm.

I NF-κB ratio by NF for control and RhoA knockdown cells (± SD). *n* = 230 ± 90 cells/bin. *P <* *0.01 (Student's *t*-test).

Binning all single cells by NF shows the relationship between the extent of cell–cell contact and nuclear TF localization (Fig5B–E). In MCF10A cells, NF-κB and YAP ratios were negatively correlated with NF in both low-density and high-density wells (Fig5B and C). Importantly, there was no significant difference in average NF-κB ratio for cells with comparable NF between low- and high-density wells (Fig5B). Average YAP ratios, however, were significantly lower in high NF cells (> 0.9) in high-density wells (Fig5C). MCF10A cells continue to divide upon reaching confluence, which results in reduced spreading and cell–ECM contact. Consistent with previous reports (Aragona *et al*, 2013), these data indicate that nuclear YAP continued to decrease after confluence as cells became more tightly packed (Supplementary Fig S4B and C). Cell–cell contact therefore suppressed nuclear localization of both NF-κB and YAP, but YAP was more sensitive to cell density.

Bayesian networks detected NF-κB dependency on NF in non-tumor MCF10A (genetic subtype Basal B, morphological group L/B), MCF7 (Luminal, L1), SUM159 (Basal B, L/B), and MDA-MB-231 (Basal B, B) cells, but not in HCC1954 (Basal A, L1) and AU565 (Luminal, L2) cells stimulated with TNFα (Fig2D). Indeed, NF-κB ratios were inversely correlated with NF in the former but not the latter cell lines (Supplementary Fig S5D and F), and the strength of the relationship (the slope of the regression line between NF-κB ratio and NF) was lower in AU565 and HCC1954 cells (Fig5D). YAP ratios, on the other hand, did decrease with NF in these cell lines (Fig5E and Supplementary Fig S5E and F). These findings confirm that NF-κB regulation was uncoupled from cell–cell contact in AU565 and HCC1954 cells, as predicted by network models, whereas YAP localization remained sensitive to cell density in these lines.

MCF7 was the only one of these lines in which no YAP ratio dependency on NF was detected. Although a negative linear correlation was observed between YAP ratio and NF in single MCF7 cells (Supplementary Fig S5E), the strength of the relationship was low (Fig5E). These data indicate that Bayesian network models identified only the most robust relationships between features and also suggest that YAP regulation by cell–cell contact may be abnormal in MCF7 cells.

### NF-κB sensitivity to cell density requires RhoA and ROCK

Because NF-κB nuclear localization was higher in MCF10A cells with few neighbors, we next asked whether inducing an ‘edge’ morphology in MCF10A cells affected NF-κB ratios and, if so, whether this effect was mediated through cytoskeletal tension. TNFα was added immediately after scrape wounding confluent monolayers of cells starting from 8 h before fixation. At all time points, edge cells showed higher NF-κB ratios than non-edge cells (Fig5F). Pre-treatment with the ROCK inhibitor Y27, however, reduced the difference in NF-κB ratios between edge and non-edge cells, especially at 1 h (Fig5G). As before, Y27-treated cells had a higher first peak of nuclear NF-κB ratios than controls (0.51 vs 0.36 for edge cells, 0.49 vs 0.3 for non-edge cells) and caused a steeper decrease in NF-κB ratio from 1 to 2 h in edge cells (24% vs 11%). Importantly, Y27 treatment eliminated the difference between edge and non-edge cells at 1 h.

To confirm that the effect of cell density on NF-κB required Rho-ROCK signaling, we knocked down RhoA in MCF10A cells and repeated the plating density experiment. RhoA knockdown (k/d) cells had an elongated morphology similar to Y27-treated cells (Fig5H) and significantly higher NF-κB ratios than control cells at all NF values (*P *<* *0.01) and (Fig5I), and NF-κB was less sensitive to NF in RhoA-depleted cells (slope of linear regression: control = 0.21, RhoA k/d = −0.05). RhoA knockdown also eliminated the NF dependency observed in wild-type MDA-MB-231 (Basal B, B) and SUM159 (Basal B, L/B) cells stimulated with TNFα (1 h) (Supplementary Fig S5G). However, NF-κB ratio dependency on ruffliness was observed in these lines (confidence > 0.9), as well as in RhoA-depleted AU565 (Luminal, L2) and T47D (Luminal, L1) cells, in which NF-κB was not connected to NF. Taken together, these data suggest that the RhoA-ROCK pathway is involved in mediating the negative regulation of NF-κB by cell–cell contact and that this link may be broken in some cancer cells.

### NF-κB is sensitive to substrate stiffness

Based on these findings, we hypothesized that cortical F-actin could couple NF-κB activation to protrusion and spreading. Specifically, nuclear translocation could be enhanced in cells that are under low cortical tension, such as protrusive cells or cells at the edge of a colony, and suppressed in round or non-protrusive cells with high cortical tension (Thoumine *et al*, 1999; Maddox & Burridge, 2003). To test this hypothesis, we cultured MCF10A and MDA-MB-231 cells on fibronectin-coated glass or flexible polyacrylamide (PA) gels with Young's moduli (a measure of stiffness) of 35 and 16 kPa (Tse & Engler, 2010) and measured cell shape and NF-κB localization in cells stimulated with TNFα. PA gels have long been used to fabricate substrates with more physiologically relevant elasticity than glass (Pelham & Wang, 1997). Substrate stiffness can control differentiation, motility, and cytoskeleton organization as cells sense and respond to matrix compliance (reviewed by Discher *et al*, 2005). Moreover, cells can tune their internal stiffness to match that of the extracellular matrix by controlling F-actin cross-linking and contraction (Solon *et al*, 2007).

**Figure 6:**
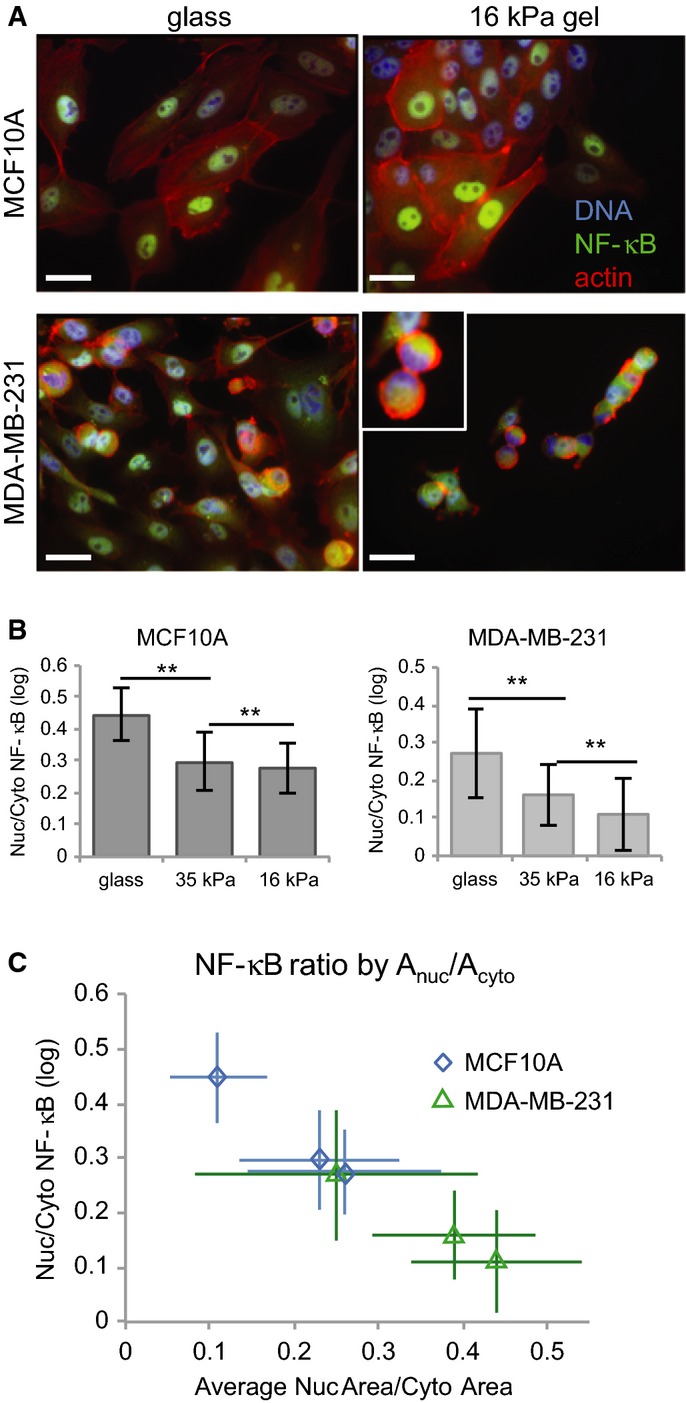
Substrate stiffness affects NF-κB activation together with cell spreading

Substrate flexibility altered morphology and NF-κB localization in both cell lines. MCF10A cells adhered and spread on PA gels but were significantly smaller in area and in tighter colonies than cells on glass (*P *<* *0.01) (Supplementary Fig S6A). Nuclear area decreased in MCF10A cells only on the most flexible (16 kPa) gels (*P *<* *0.01) (Supplementary Fig S6B). MDA-MB-231 morphology was extremely sensitive to substrate flexibility. Cells on glass ranged from large and flat to small and round, whereas cells spread poorly on 35 kPa gels and hardly at all on 16 kPa gels, indicating high levels of actin contractility and/or poor ECM adhesion. Cell and nucleus area decreased with stiffness (Supplementary Fig S6C and D), and nuclei in round, poorly spread cells were often crescent or cup shaped (Fig6A, inset). NF-κB ratios decreased dramatically with substrate stiffness in both cell lines (Fig6B). In fact, about 5% of MDA-MB-231 cells on 16 kPa gels had negative log ratios, indicating little or no NF-κB activation. NF-κB ratios were also strongly associated with A_nuc_/A_cyto_ (*R*^2^ = 0.98) (Fig6C). Because cell spreading represents the balance between internal contractile forces and external resistance, these data indicate that NF-κB regulation is sensitive to cytoskeletal tension.

Representative images of TNFα-stimulated MCF10A (top) and MDA-MB-231 (bottom) cells on fibronectin-coated glass (left) and 16-kPa PA gels (right). Inset: crescent-shaped nuclei. Scale bar = 20 μm.

Average NF-κB ratios for cells on each substrate (± SD). MCF10A, *n* = 1,215 ± 660 cells/substrate. MDA-MB-231, *n* = 1,440 ± 364 cells/substrate. **P <* *0.001 (Student's *t*-test).

Average NF-κB ratio by A_nuc_/A_cyto_ (± SD).

### N-cadherin depletion induces changes in cell shape and NF-κB localization

**Figure 7:**
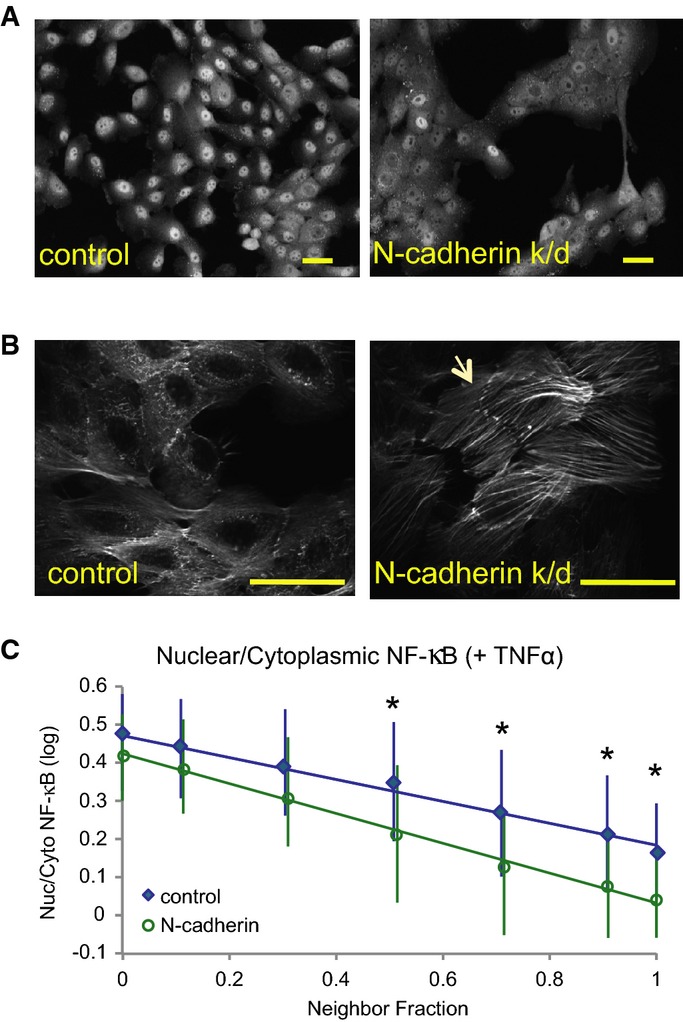
Effects of N-cadherin depletion on cell shape and NF-κB activation

Finally, we asked whether N-cadherin expression, which was associated with mesenchymal-like morphology and high NF-κB activation, affected NF-κB localization. MCF10A cells were transfected with siRNA against N-cadherin or mock-transfected (control) and seeded at varying densities. N-cadherin, but not E-cadherin, levels were significantly reduced after 3 days (*P *<* *0.01) (Supplementary Fig S7A and B), and N-cadherin depletion resulted in a dramatic change in cell morphology (Fig7A). Cells lacking N-cadherin grew exclusively in colonies, so NF was significantly higher in N-cadherin k/d wells than in control wells of comparable density (0.88 ± 0.05 vs 0.58 ± 0.04 for medium-density wells; *P *<* *0.01) (Supplementary Fig S7C). Nuclear area and A_nuc_/A_cyto_ were also significantly higher in N-cadherin knockdowns (*P *<* *0.01) (Supplementary Fig S7D and E). N-cadherin depleted cells had strikingly thick F-actin fibers, many of which terminated at cell membranes and appeared to be contiguous with fibers in adjacent cells (Fig7B, arrow). NF-κB ratios were significantly lower in TNFα-stimulated N-cadherin k/d cells with NF ≥ 0.5, i.e. non-edge cells (Fig7C). These data suggest that N-cadherin may modulate NF-κB activation in response to cytokine stimulation via effects on cell shape and cytoskeletal organization.

Representative images of control and N-cadherin knockdown cells stained for NF-κB. Scale bar = 20 μm.

High-magnification (60×) images of F-actin in control and knockdown cells. Arrow indicates F-actin bundles that appear to terminate at cell–cell adhesions. Scale bar = 25 μm.

Nuclear/cytoplasmic NF-κB ratios by neighbor fraction (binned) after TNFα stimulation (mean ± SD). Control, *n* = 4,920 ± 4,880 cells/bin. N-cadherin k/d, *n* = 4,817 ± 6,300 cells/bin. *P <* *0.01 (Student's *t*-test).

## Discussion

Cell shape, in particular the cortical cytoskeleton and curvature of the plasma membrane, has been described as a ‘repository of information’ that can modulate signal transduction (Rangamani *et al*, 2013). We speculated that shape variation could account for some of the heterogeneity observed in NF-κB localization in breast cancer cells. We exploited the naturally occurring variation in cellular populations to uncover relationships between morphology and transcription factor activation in breast tumor and non-tumor cell lines, and tested model predictions using different techniques. To our knowledge, this is the first time that cell shape and context have been extensively quantified and linked to NF-κB regulation in breast cancer cells.

The effects of cell shape on NF-κB signaling were revealed through Bayesian analysis of single-cell datasets. In addition to NF-κB, Bayesian network models identified dependencies between morphological features and nuclear localization of YAP, a well-known shape-regulated TF (Dupont *et al*, 2011; Aragona *et al*, 2013). In contrast, Jun activation downstream of TNFα was not dependent on cell shape, which suggests that the dependencies we identified were specific to the NF-κB pathway and did not simply scale with differences in TNFR ligation. Whether shape has a role to play in regulating the activity of other TFs remains unclear. We speculate that TFs whose function is regulated largely through subcellular localization (i.e., are inhibited through cytoplasmic or membrane sequestration) are likely to be influenced by cell shape, which would couple transcriptional outputs to morphological inputs. In contrast, TFs that are constitutively localized to the nucleus and are regulated transcriptionally or via phosphorylation may be less dependent on morphological cues.

Well-spread, protrusive cells with few cell–cell contacts (mesenchymal-type morphologies) tended to be more responsive to TNFα than cells that were poorly spread and/or had many neighbors (epithelial-type morphologies). Because NF-κB drives expression of EMT genes, we propose that this difference in responsiveness could have the effect of reinforcing mesenchymal morphology in cancer cells, thus generating a positive feedback loop. In support of this model, chemically, physically, or genetically inducing cells to adopt mesenchymal-like shapes resulted in greater NF-κB activation. Conversely, inducing cell rounding and/or strengthening cell–cell adhesions reduced NF-κB nuclear localization. Furthermore, expression of N-cadherin, an EMT marker (Andrews *et al*, 2012), was associated with mesenchymal-like morphology and high NF-κB activation in cancer lines, and its depletion induced an epithelial-like phenotype and suppressed NF-κB activation in MCF10A cells.

Bayesian networks predicted that NF-κB was decoupled from cell–cell contact after stimulation with TNFα in the tumor breast cell lines HCC1954 and AU565, and these results were confirmed by forcing cells into high and low NF by altering plating densities. This suggests that TF regulation in some cancer cells might become insensitive to cell morphology and context, which would result in inappropriate proliferation, survival, or migration. More work is needed to investigate how such decoupling might occur and what its consequences might be in the context of cancer.

Our data point to a critical role for cortical actomyosin tension in NF-κB regulation. Spreading cells and protrusive cells at the edge of a scrape wound experience a decrease in cortical tension, whereas cells rounding up due to loss of cell–matrix adhesion, entry into mitosis, or MT depolymerization have stiff actin cortices (Thoumine *et al*, 1999; Maddox & Burridge, 2003). We observed that cells with few neighbors, cells at wound edges, and cells in which RhoA-ROCK-myosin II signaling was blocked had increased levels of nuclear NF-κB in response to TNFα. On the other hand, cells with rounded morphologies induced by nocodazole or taxol treatment, flexible substrates, or high plating density had less nuclear NF-κB. In addition, the difference in NF-κB activation between wound edge and non-edge cells was lost when actomyosin-mediated contractility was suppressed by ROCK inhibition, and the suppressive effect of high neighbor fraction was lost in the absence of RhoA.

Although I-κB is reported to bind to dynein (Crepieux *et al*, 1997) and MTs (Chi *et al*, 2012), our observations do not suggest that NF-κB translocation depends on MT-mediated transport. Treating cells with Noc and Y27 together shifted both cell shape and NF-κB ratios toward control values, and stabilizing MTs with taxol induced both rounded morphology and low NF-κB activation. Instead, we propose that MT depolymerization suppresses NF-κB by increasing cortical tension. Dynamic MTs provide mechanical resistance to actomyosin tension at the membrane (Wang *et al*, 2001), and Noc treatment can induce RhoA activation by releasing its activator GEF-H1 (Chang *et al*, 2008). Thus, the effects of ROCK inhibitors and Noc could cancel each other out. These results may help explain conflicting reports regarding the role of the cytoskeleton in NF-κB regulation, as they imply that drug effects depend on cell tension and shape (Rosette & Karin, 1995; Nemeth *et al*, 2004; Mackenzie & Oteiza, 2006; Ishihara *et al*, 2013). Drug effects may also depend on which NF-κB pathways are activated. We found that low doses of nocodazole increased NF-κB nuclear localization in unstimulated cells (Supplementary Fig S4A), similar to Rosette and Karin (1995). This effect could be due to the induction of cellular stress responses to drug treatment. Cytoskeleton-modifying drugs affected cancer cell lines to different degrees, but the changes in cell shape they caused were consistently predictive of changes in NF-κB ratio (Fig4D).

Further support for this model comes from the effects of N-cadherin depletion on cell shape and NF-κB in MCF10A cells. N-cadherin adhesions are less resistant to pulling forces than E-cadherin adhesions (Chu *et al*, 2006), so cells expressing only E-cadherin would exert more tensile force on one another. N-cadherin k/d cells are therefore predicted to be in a state of extremely high tension, and this is evident from the presence of thick F-actin fibers terminating at cell–cell contact points (Fig 7B, arrow). If actomyosin tension suppresses NF-κB activation, we would predict that N-cadherin k/d cells in the middle of colonies, with extensive cell–cell contacts (high NF), would have even lower NF-κB ratios than control cells, and in fact, this is what we observed. N-cadherin depleted cells also had significantly larger nuclei than controls, which could also be due to actin-mediated connections between the nucleus and cell–cell/cell–matrix adhesions (Khatau *et al*, 2009). Moreover, NF-κB ratio in TNFα-stimulated cells was dependent on nuclear area in 15/19 cell lines, including MCF10A (Fig2). Thus, N-cadherin expression could enhance NF-κB activation in cancer cells through its effects on cell morphology.

Altering cell shape with cytoskeleton-modifying drugs also affected the synchronicity and duration of NF-κB cycling in and out of the nucleus. Heterogeneity in nuclear-cytoplasmic shuttling may arise from differences in negative feedback caused by I-κB transcription, IKK activation, or the availability of NF-κB (Kearns *et al*, 2006; Ashall *et al*, 2009; Paszek *et al*, 2010; Kalita *et al*, 2011), and our data suggest that cell shape is also an important source of cell-to-cell variation. Heterogeneity may have evolved to ensure population robustness and reduce tissue sensitivity to inflammatory signals (Paszek *et al*, 2010). We speculate that shape-mediated differences in NF-κB shuttling could therefore have profound effects on how healthy, wounded, and pathological tissues respond to cytokines. While some models of oscillation have been proposed which take morphology into account (Terry & Chaplain, 2011; Sturrock *et al*, 2012), more work is needed to determine how cell shape impacts NF-κB cycling. Further high-content studies that incorporate live cell GFP-p65 and shape measurements will overcome the acyclic nature of Bayesian networks and elucidate whether a feedback exists from NF-κB to cell shape and provide insight into these mechanisms.

This work demonstrates the key role of shape and the microenvironment in regulating signal transduction and gene expression. Shape-dependent regulation of NF-κB may have evolved by facilitating the ability of isogenic cell types to achieve different responses to a uniform signal. For example, this mechanism could enable an inflammatory response in cells at a wound site while limiting the response in cells distal to the wound. The effects of cell shape on NF-κB signaling could also drive metastatic processes if cells at the invasive edge of tumors are more likely to activate NF-κB in response to inflammatory cytokines such as TNFα. Decoupling of NF-κB from the cell context and morphology could also drive tumorigenesis through increasing survival and proliferation.

Finally, these studies illustrate the utility of Bayesian network modeling for uncovering complex relationships between cell form and function, and they highlight the importance of context in determining cell behavior. Cellular context was shown to be an important source of variation in virus infectivity and endocytosis, and accounting for such differences in cellular states could explain much of the heterogeneity observed within cellular populations (Snijder *et al*, 2009). These findings indicate that cell shape and context are also important determinants of transcription factor regulation and cells’ response to chemical signals. Thus, care should be taken to consider factors such as density when interpreting data from experiments where culture conditions may vary, including RNAi screens, drug screens, and comparisons between cell lines.

## Materials and Methods

### Cell culture and staining

All cancer cell lines were grown in DMEM:F12 (Gibco) plus 5% heat-inactivated FBS (Gibco). MCF10A cells were maintained in DMEM:F12 medium (Gibco) supplemented with 100 ng/ml EGF, insulin, 0.5 mg/ml hydrocortisone, and cholera toxin (Sigma). Imaging experiments were performed in 384-well Cell Carrier plates (Greiner) on cells cultured for 3 days unless otherwise specified. For the initial screen, 1,000 cells/well were seeded in 14 replicate wells per cell line per plate. Live cell imaging was performed using a climate control chamber (37°C, 5% CO_2_, 70% humidity).

Cells were labeled with 10 μM DHE (Invitrogen) 30 min prior to fixation in 4% PFA. Cells were permeabilized in 0.1% Triton X-100 and stained with anti-p65 antibody (Abcam), secondary antibody (Alexa488 or Alex647 anti-rabbit, Invitrogen) and DAPI or Hoechst. In later experiments, segmentation of cell bodies was performed using anti-p65 or mouse anti-YAP (Santa Cruz) labels. Rabbit phospho-c-Jun (Ser63) antibody was from New England Biolabs. Mouse anti-N-cadherin was from Abcam.

### Cytokine, drug treatments, and transfection

Human recombinant TNFα (Life Technologies) was added to a final concentration of 10 ng/ml. Y-27632 (Sigma) was used at 10 μM unless otherwise specified, and nocodazole was used at 0.1 μg/ml unless otherwise specified. Blebbistatin, taxol, and DMSO were obtained from Sigma. H1152 was from Tocris Bioscience. Plasmid transfection with GFP-p65/RelA (Addgene; plasmid ID 23255; (Chen *et al*, 2001)) was performed using Lipofectamine 2000 (Invitrogen), and GFP-positive cells were harvested by FACS 24 h before imaging. Reverse siRNA transfection was performed using Lipofectamine RNAiMAX (Invitrogen) and RhoA (siGENOME SMARTpool, Dharmacon) and N-cadherin (ON-Target Plus SMARTpool, Dharmacon) siRNA. See also Supplementary Materials and Methods.

### High-content image acquisition and analysis

Image acquisition was performed using an Opera Cell::Explorer-automated spinning disk confocal microscope. The initial screen was performed using a 20× air objective lens (NA = 0.45) (PerkinElmer), and 12–30 fields of view were imaged in each well. Subsequent experiments were imaged using a 20× water objective lens (NA = 0.6). Cell segmentation was performed using Acapella software (PerkinElmer). See Supplementary Materials and Methods for details of cell segmentation and feature extraction.

### Live cell imaging

Cells were transfected with GFP-p65 plasmid for 5 h using Lipofectamine 2000 (Invitrogen), and cells expressing low to moderate levels of GFP were sorted by flow cytometry (FACSAria) and seeded in 384-well plates. TNFα (10 ng/ml) was added just prior to the start of movies (∽1 min). Image acquisition was performed using an Opera-automated spinning disk confocal microscope equipped with climate control (37°, 5% CO_2_, 79% humidity) and 20× water lens, and 8 fields of view were imaged in replicate wells for each condition. Quantification of GFP-p65 intensity was measured in nuclear and perinuclear regions using ImageJ. The same corrections (range and gamma) were applied to all raw images in each figure using Columbus analysis software (PerkinElmer) or Photoshop (Adobe).

### Plating density and scrape wound assays

Cells were seeded at 500, 1,000, 2,000, and 4,000 cells/well for NF-κB/YAP density experiments and 500–8,000 cells/well for cadherin and RhoA knockdown experiments, and all cells were cultured for 3 days before fixing and staining. Scrape wounds were made using a pipet tip in confluent monolayers of cells cultured for 3 days; then, medium was removed and replaced with fresh medium containing TNFα at the indicated times before fixation.

### Polyacrylamide gel fabrication and imaging

Flexible substrates were made by casting solutions containing different concentrations of acrylamide and bis-acrylamide (Sigma) as described by Tse and Engler (2010) onto glass coverslips functionalized with NaOH followed by APTMS and glutaraldehyde (Sigma). Cured gels were incubated with 25 μM sulfo-SANPAH (Thermo-Pierce) in 50 mM HEPES (pH 8.5) under UV light for 10 min, washed, and incubated with 50 μg/ml fibronectin (Sigma) in PBS overnight at 4°C. Glass coverslips were also coated with 50 μg/ml fibronectin. Cells were plated on PA gels for 3 days, fixed and stained, mounted using Fluoromount G (Southern Biotech), and imaged using a Nikon Eclipse epifluorescence microscope (20×). Analysis was performed using Columbus software (PerkinElmer).

### Wavelet analysis

We extracted the characteristic periods and amplitudes from the oscillation data at 5-min intervals using the WAVOS Matlab toolbox (Harang *et al*, 2012).

### PCA, clustering, and statistical tests

Principal component analysis was carried out using Cluster 3.0 and MATLAB software based on Z-scores [(value-mean)/SD]. Hierarchical clustering was performed with Cluster 3.0 and visualized using Java Tree View. *P* values were determined using Student's *t*-test and ANOVA (Excel and MATLAB). R and R^2^ values were determined using Excel or MATLAB (Pearson correlation unless otherwise specified).

### Bayesian network and multivariate linear regression modeling

See Supplementary Materials and Methods for details and methods.

### Data availability

Single cell data used to generate Bayesian network models for 19 cell lines ± TNFα (Supplementary Dataset S1), explanation of morphological features (Supplementary Dataset S2), and data used for multivariate linear regression (fold change compared to control for each cell line) (Supplementary Dataset S3) are provided as Supplementary Datasets S1, S2, and S3.

Image datasets for the cell lines used for morphological profiling are available from DRYAD: http://dx.doi.org/10.5061/dryad.tc5g4.

## Supporting Information

Supplementary Movies

Review Process File
