# Supplementary figures and images for: Cell shape and the microenvironment regulate nuclear translocation of NF-κB in breast epithelial and tumor cells
Source: Mol Syst Biol. 2015 Mar 3;11(3):0790. doi: 10.15252/msb.20145644 (PMC4380925; doi:10.15252/msb.20145644)

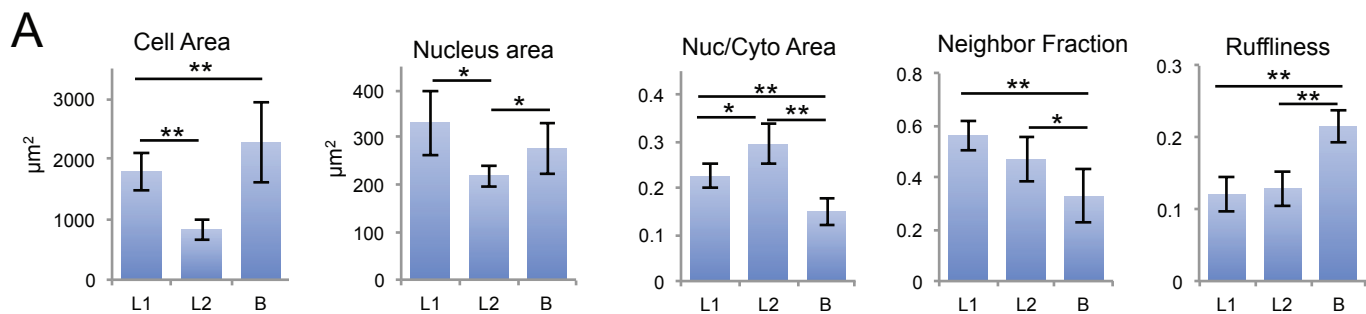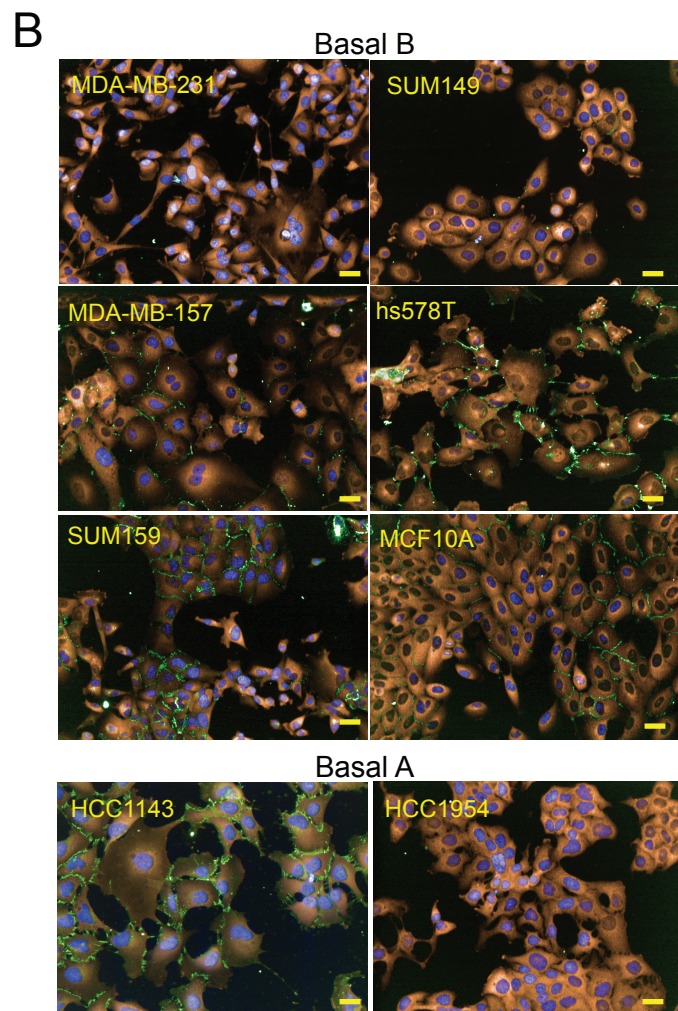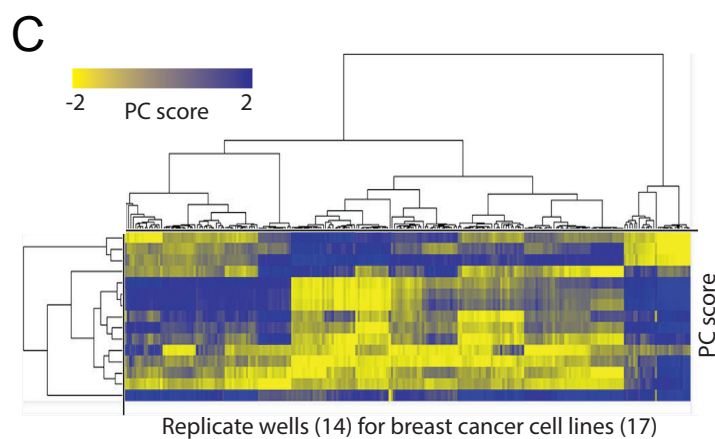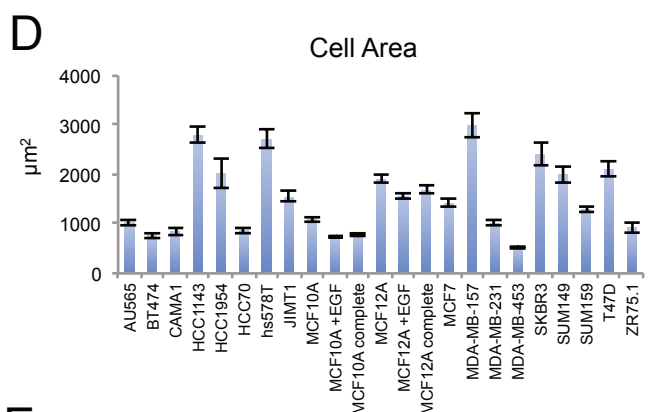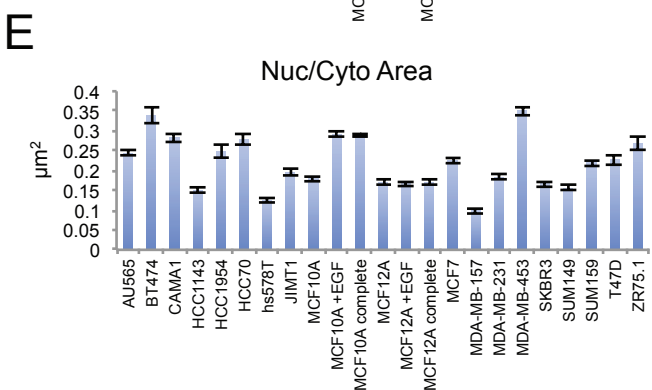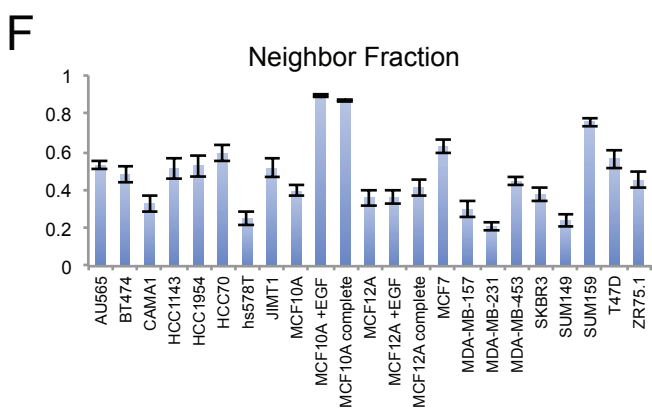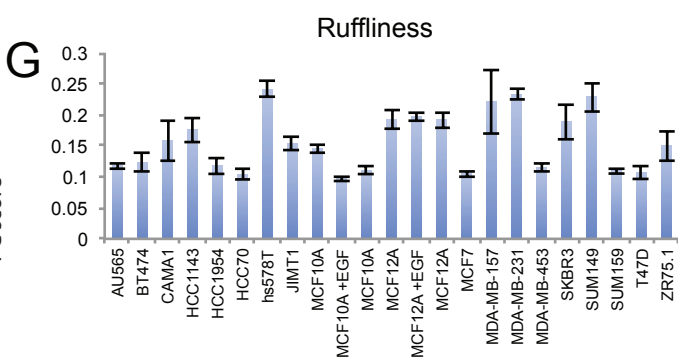

Supplement: Supplementary file 1 [file msb0011-0790-sd1.pdf]

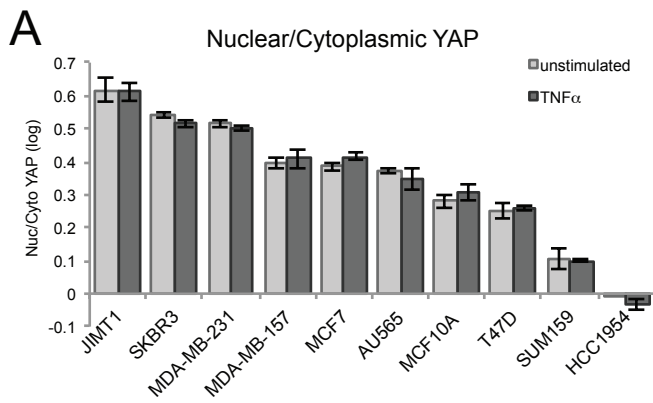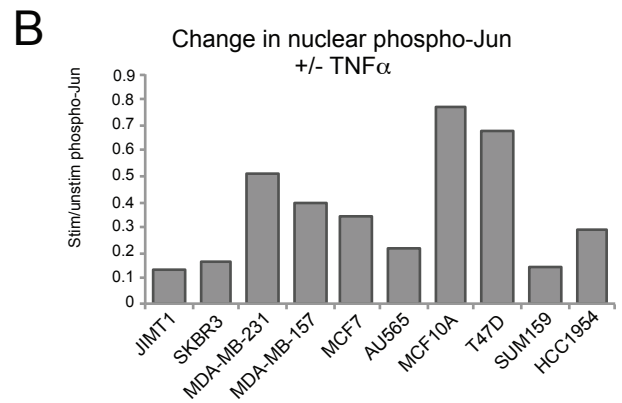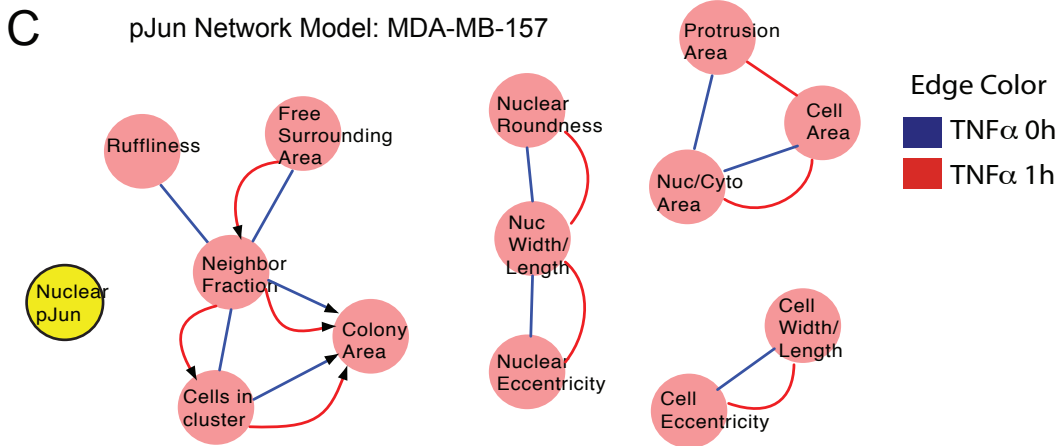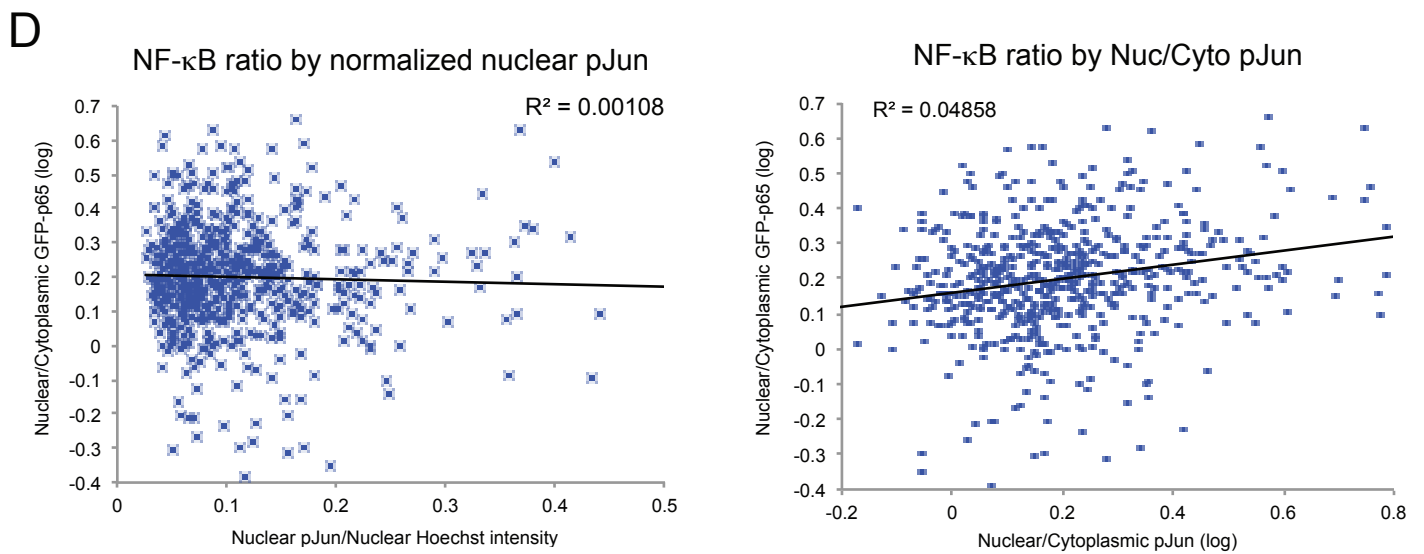

Supplement: Supplementary file 3 [file msb0011-0790-sd3.pdf]

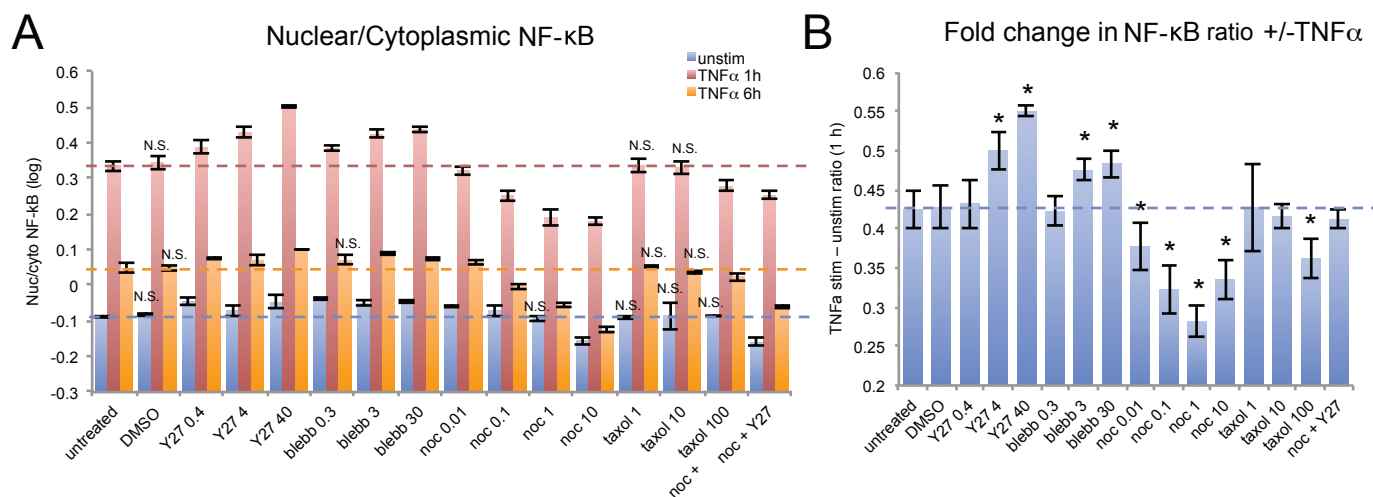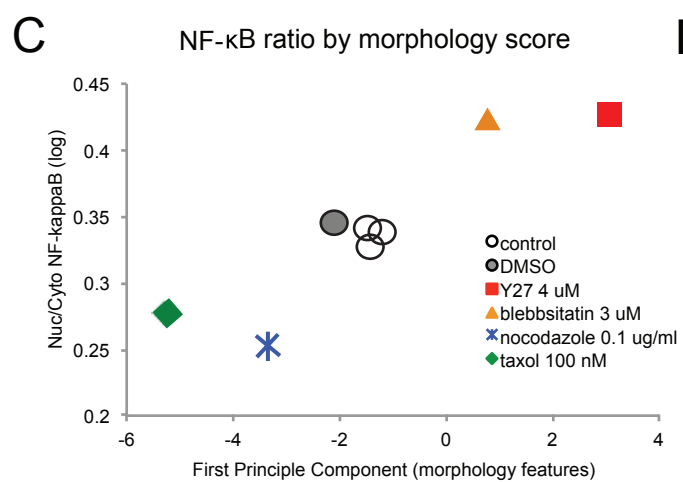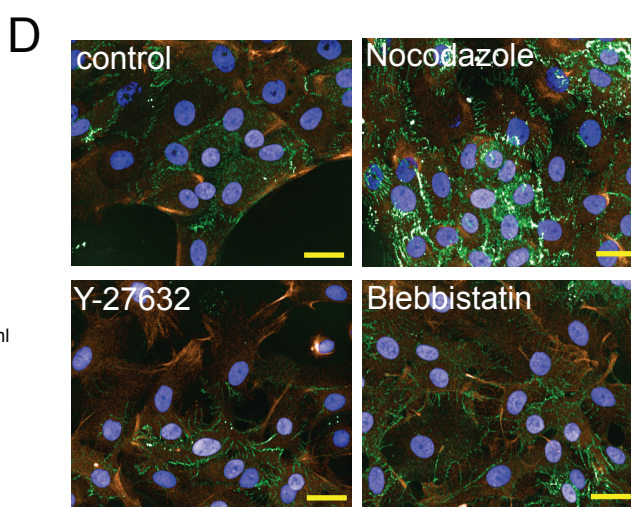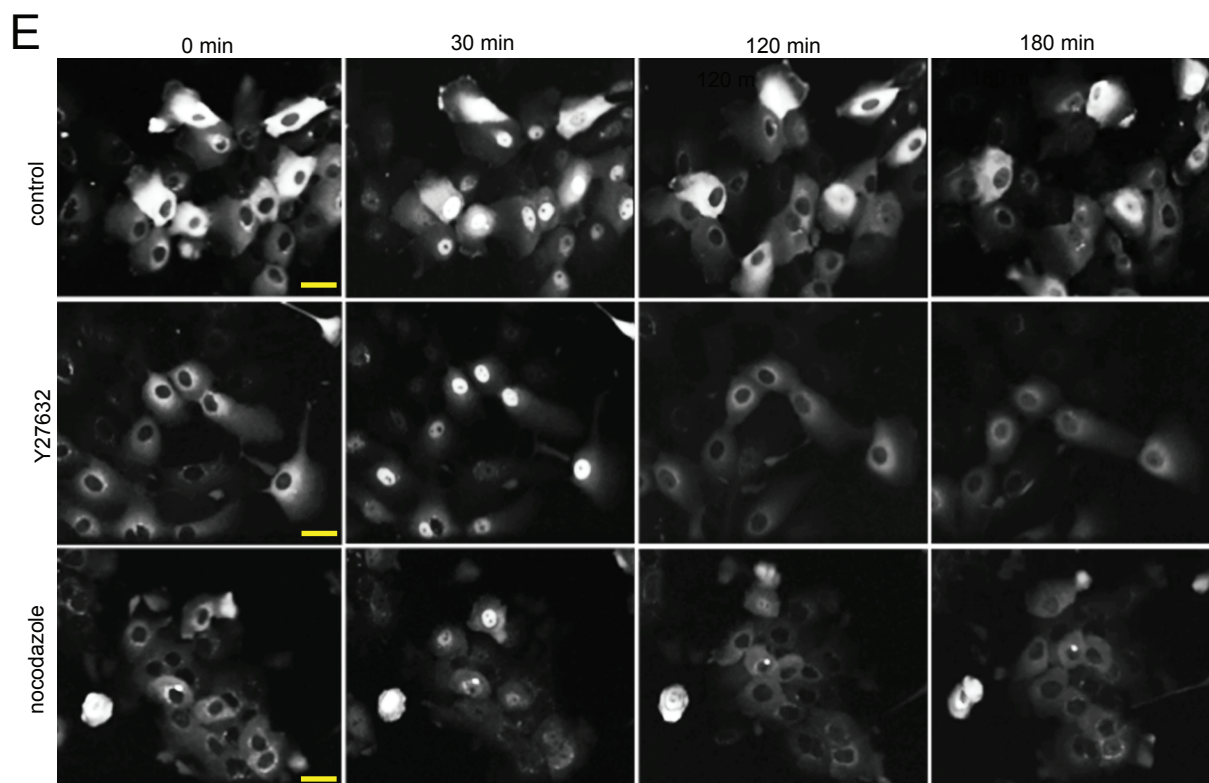

Supplement: Supplementary file 4 [file msb0011-0790-sd4.pdf]

**A**

PA gels: Cell Area

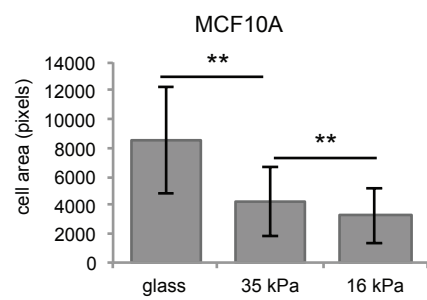**B**

PA gels: Nucleus Area

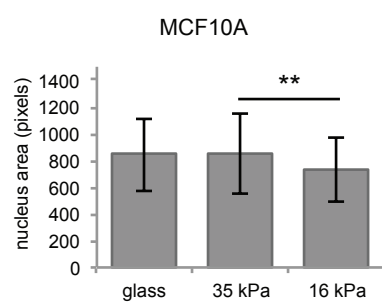**C**

PA gels: Cell Area

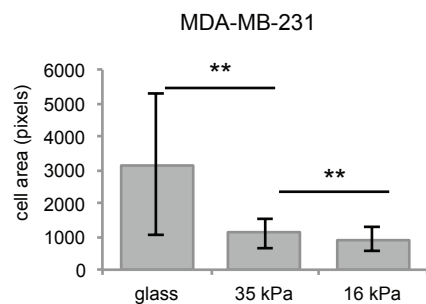**D**

PA gels: Nucleus Area

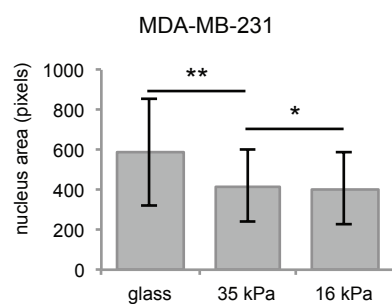

Supplement: Supplementary file 6 [file msb0011-0790-sd6.pdf]

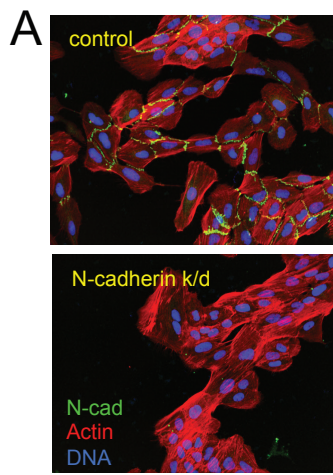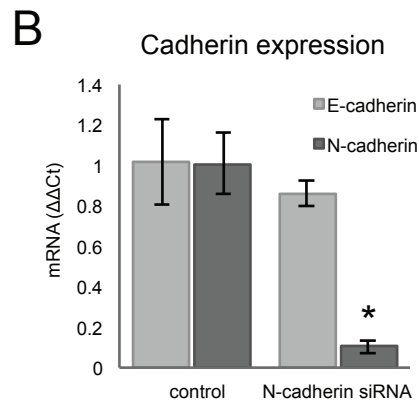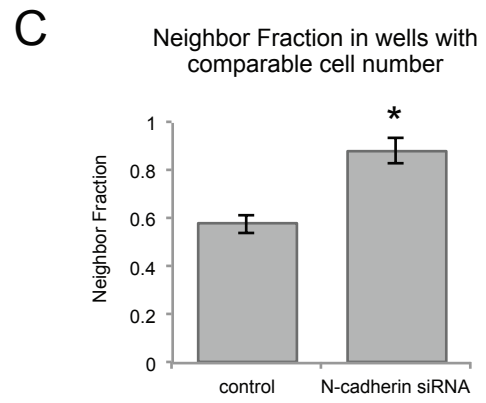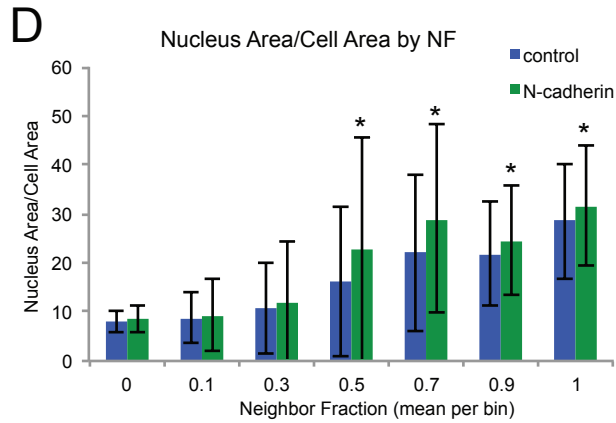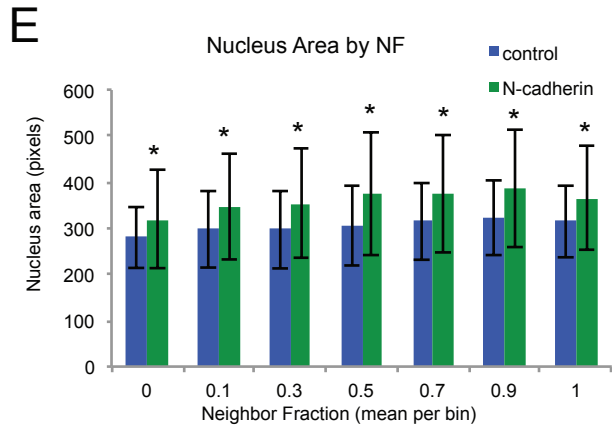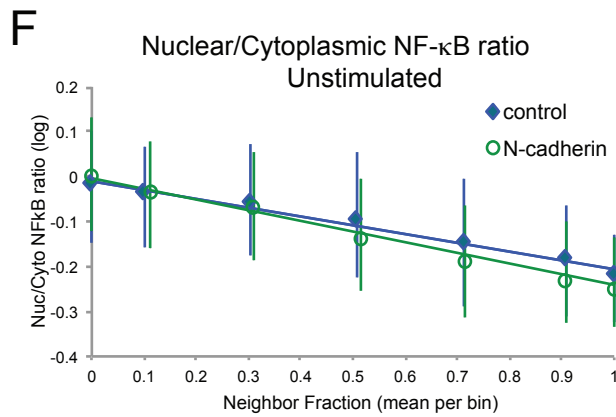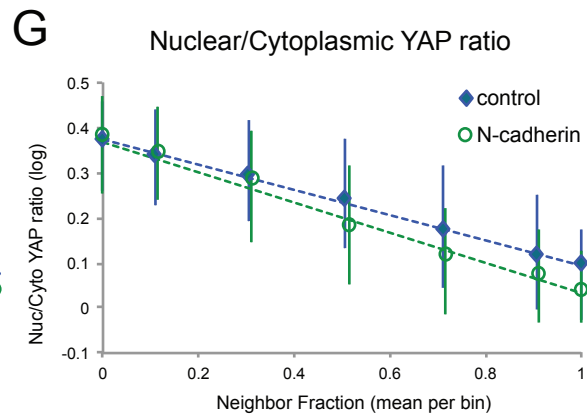

Supplement: Supplementary file 7 [file msb0011-0790-sd7.pdf]
