# Supplementary material for: Cell shape and the microenvironment regulate nuclear translocation of NF-κB in breast epithelial and tumor cells
Source: Mol Syst Biol. 2015 Mar 3;11(3):0790. doi: 10.15252/msb.20145644 (PMC4380925; doi:10.15252/msb.20145644)
Supplement: Supplementary file 2 [file msb0011-0790-sd2.pdf]

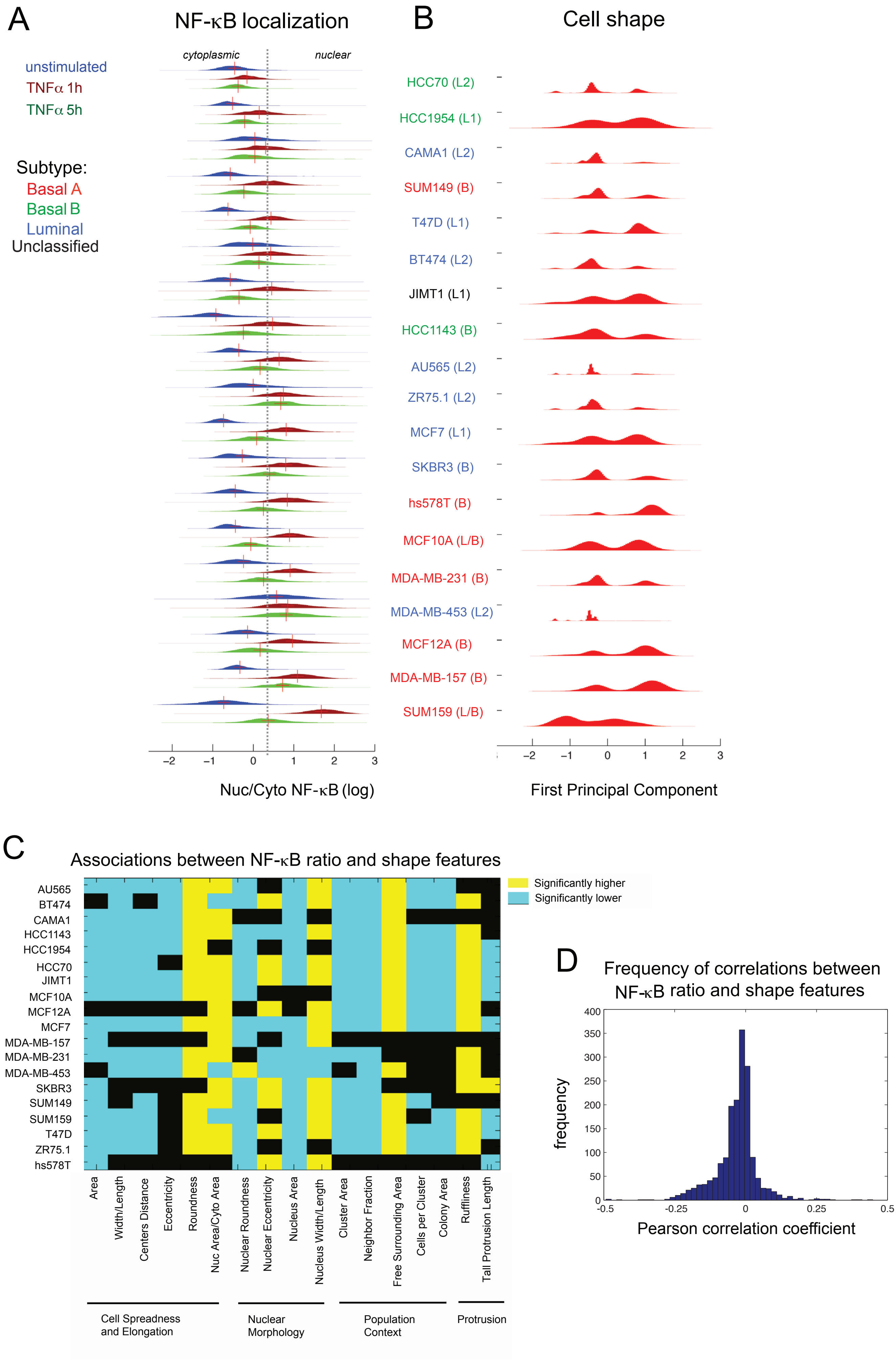

A
NF-κB localization
cytoplasmic
nuclear
unstimulated
TNFα 1h
TNFα 5h
Subtype:
Basal A
Basal B
Luminal
Unclassified
Nuc/Cyto NF-κB (log)
B
Cell shape
HCC70 (L2)
HCC1954 (L1)
CAMA1 (L2)
SUM149 (B)
T47D (L1)
BT474 (L2)
JIMT1 (L1)
HCC1143 (B)
AU565 (L2)
ZR75.1 (L2)
MCF7 (L1)
SKBR3 (B)
hs578T (B)
MCF10A (L/B)
MDA-MB-231 (B)
MDA-MB-453 (L2)
MCF12A (B)
MDA-MB-157 (B)
SUM159 (L/B)
First Principal Component
C
Associations between NF-κB ratio and shape features
Significantly higher
Significantly lower
AU565
BT474
CAMA1
HCC1143
HCC1954
HCC70
JIMT1
MCF10A
MCF12A
MCF7
MDA-MB-157
MDA-MB-231
MDA-MB-453
SKBR3
SUM149
SUM159
T47D
ZR75.1
hs578T
Area
Width/Length
Centers Distance
Eccentricity
Roundness
Nuc Area/Cyto Area
Nuclear Roundness
Nuclear Eccentricity
Nucleus Area
Nucleus Width/Length
Cluster Area
Neighbor Fraction
Free Surrounding Area
Cells per Cluster
Colony Area
Ruffliness
Tall Protrusion Length
Cell Spreadness and Elongation
Nuclear Morphology
Population Context
Protrusion
D
Frequency of correlations between NF-κB ratio and shape features
frequency
Pearson correlation coefficient
