# Supplementary material for: Cell shape and the microenvironment regulate nuclear translocation of NF-κB in breast epithelial and tumor cells
Source: Mol Syst Biol. 2015 Mar 3;11(3):0790. doi: 10.15252/msb.20145644 (PMC4380925; doi:10.15252/msb.20145644)

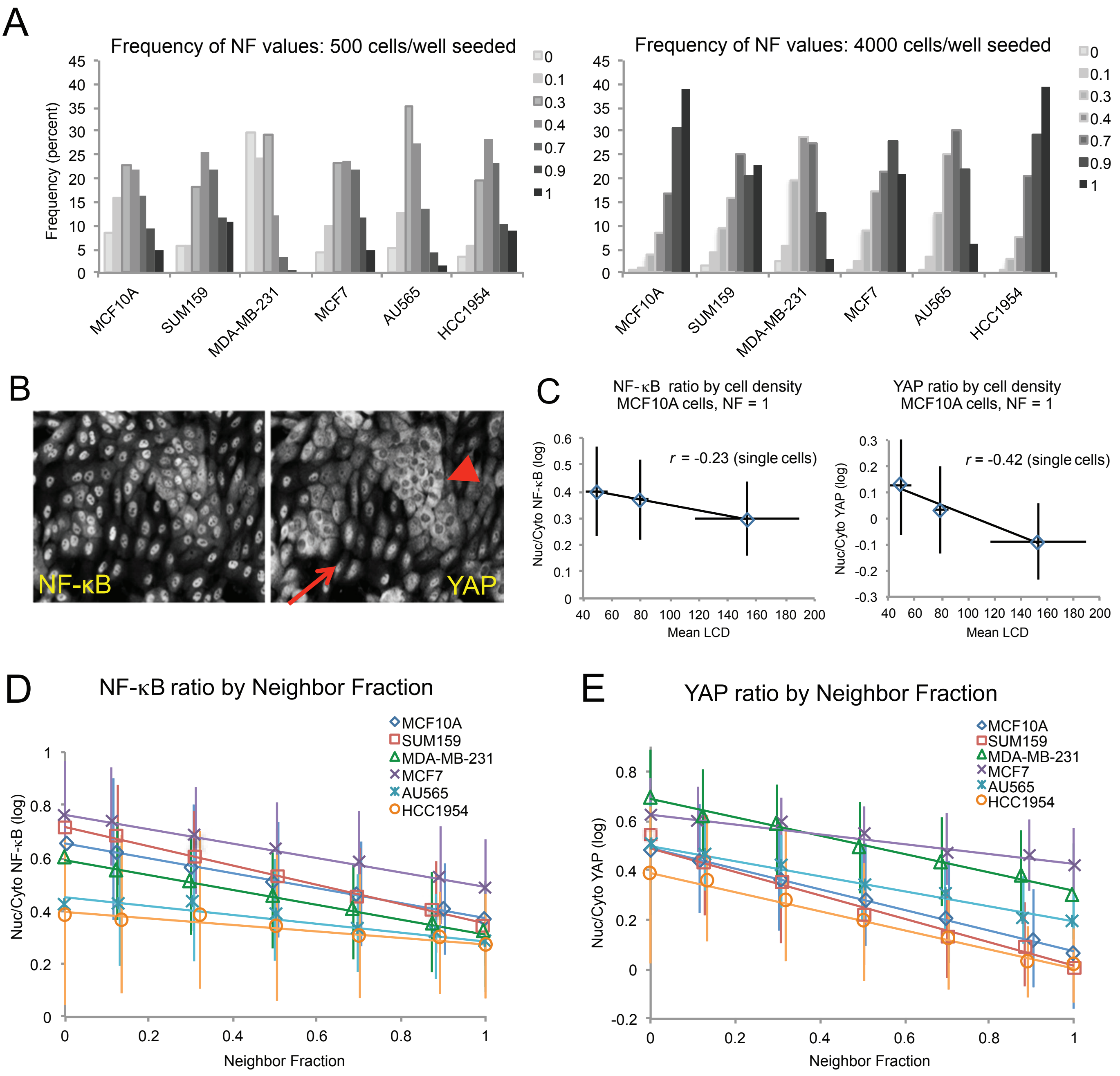

F

Correlations between NF and TF ratio in single cells

	NF-κB corr. (r)	YAP corr. (r)	cells (n)
MCF10A	-0.358	-0.467	19489
SUM159	-0.454	-0.547	6005
MDA-MB-231	-0.303	-0.382	10379
MCF7	-0.345	-0.353	9170
AU565	-0.130	-0.399	7806
HCC1954	-0.206	-0.367	5684

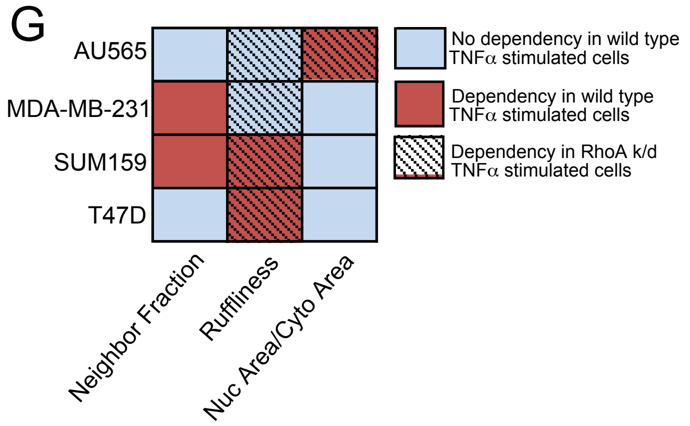

Supplement: Supplementary file 5 [file msb0011-0790-sd5.pdf]
