# Supplementary material for: Cell shape and the microenvironment regulate nuclear translocation of NF-κB in breast epithelial and tumor cells
Source: Mol Syst Biol. 2015 Mar 3;11(3):0790. doi: 10.15252/msb.20145644 (PMC4380925; doi:10.15252/msb.20145644)
Supplement: Supplementary file 8 [file msb0011-0790-sd8.docx]

Table S1. Spearman correlation coefficients between NF-κB ratios and gene expression across 18 breast cell lines

| **Condition** | **TNFRSF1A** | **IKBKE** | **NFKB1** | **NFKB2** | **NFKBIA** | **TRAF2** | **RIPK1** | **TRAF5** | **TNIK** | **TIFA** | **IKBKG** | **IKBKB** | **RELA** | **RELB** |
| --- | --- | --- | --- | --- | --- | --- | --- | --- | --- | --- | --- | --- | --- | --- |
| **unstimulated** | -0.071 | 0.005 | 0.069 | -0.038 | -0.084 | 0.426 | 0.385 | 0.102 | 0.154 | 0.005 | -0.152 | 0.104 | -0.261 | -0.166 |
| **TNFa 1h** | 0.094 | -0.158 | 0.350 | 0.063 | -0.001 | -0.230 | 0.067 | 0.288 | 0.009 | 0.069 | 0.088 | -0.278 | -0.331 | 0.170 |
| **TNFa 5h** | -0.168 | -0.077 | 0.150 | -0.179 | -0.104 | 0.296 | -0.007 | 0.298 | 0.088 | 0.170 | -0.112 | 0.042 | -0.439 | -0.358 |
