# Supplementary material for: Cell shape and the microenvironment regulate nuclear translocation of NF-κB in breast epithelial and tumor cells
Source: Mol Syst Biol. 2015 Mar 3;11(3):0790. doi: 10.15252/msb.20145644 (PMC4380925; doi:10.15252/msb.20145644)
Supplement: Supplementary file 9 [file msb0011-0790-sd9.docx]

Table S2: Cell lines used in multivariate linear regression

| **Cell Line** | **Genetic Subtype** | **Shape Cluster** |
| --- | --- | --- |
| AU565 | BaB | L2 |
| HCC1954 | BaA | L1 |
| MCF10A, base medium | BaB | L/B |
| MCF10A, +EGF | BaB | L/B |
| MCF10A, complete medium | BaB | L/B |
| MCF7 | Lu | L1 |
| MDA-MB-157 | BaB | B |
| MDA-MB-231 | BaB | B |
| SKBR3 | Lu | L1 |
| SUM159 | BaB | L/B |
| T47D | Lu | L1 |
| HeLa | - | L2 |
