# Supplementary material for: Cell shape and the microenvironment regulate nuclear translocation of NF-κB in breast epithelial and tumor cells
Source: Mol Syst Biol. 2015 Mar 3;11(3):0790. doi: 10.15252/msb.20145644 (PMC4380925; doi:10.15252/msb.20145644)
Supplement: Supplementary file 10 [file msb0011-0790-sd10.docx]

Table S3: Treatments used in multivariate linear regression

| **Drug** | **Dose: Time** |
| --- | --- |
| Y-27632 | 10 nM: 2 h, 4 h, 24 h  20 nM, 50 nM: 24 h |
| Blebbistatin | 25 uM: 2 h, 4 h, 24 h |
| nocodazole | 1 ug/ml: 2 h |
| EDTA | 0.75 mM: 2 h |
| Hydrocortisone | 0.5 mg/ml: 2 h |
| AG1478 (EGFR inhibitor) | 0.2 uM: 24 h |
