# Supplementary material for: Cell shape and the microenvironment regulate nuclear translocation of NF-κB in breast epithelial and tumor cells
Source: Mol Syst Biol. 2015 Mar 3;11(3):0790. doi: 10.15252/msb.20145644 (PMC4380925; doi:10.15252/msb.20145644)
Supplement: Supplementary file 14 [file msb0011-0790-sd14.docx]

**Supplementary Materials and Methods**

*Cell segmentation and shape feature extraction*

Nuclei were segmented using the Hoechst/DAPI channel (405/450) and then cell bodies were segmented using the DHE (488/600), NF-κB (488/570 or 640/690), or YAP channel (488/570 or 640/690), depending on the experiment. The nucleus region was eroded by one pixel in order to compensate for segmentation errors. The perinuclear region, used to measure cytoplasmic NF-κB /YAP intensity was defined as a ring 2-7 pixels from the nucleus border.

Intensities in all channels were measured in each subcellular region: nucleus, cytoplasm, ring region, membrane region (4 pixels in from cell border), and whole cell. A subtraction step was performed to compensate for DHE fluorescence in the 488/570 channel based on relative fluorescence intensity per pixel in that channel of cells labeled with DHE alone (0.18). NF-κB and YAP ratios were determined as log(Nuclear intensity/Ring region intensity). Nuclear pJun intensity was normalized to nuclear DAPI/Hoechst intensity because cytoplasmic levels of phosphorylated Jun were very low. However, the same network modeling results were obtained using the log(Nuclear/Ring region intensity) measurement for pJun.

Geometric features measured include: the area of all subcellular regions; the length, width, and elongation (length/ width) of the cell and nucleus; cell and nuclear roundness (form factor); nucleus area/cytoplasm area; protrusion area/cell area; and area, length, width, and roundness of individual protrusions. Measures of cell polarity include the angle between cell protrusions through the cell centroid, and the angle between cell major/minor axes and individual protrusion major/minor axes (where major/minor axes are determined using PCA).

Context features that describe the relationship of a cell to other cells were also measured. Neighbor fraction (NF) was determined as the proportion of a cell’s border in contact with other cells. Voronoi segmentation was used to get approximation of the free space around cells. Cell clusters were also segmented, and cluster areas and the cells’ position within colonies (edge vs. non-edge) were determined. Local Cell Density (LCD) was measured by calculating the free space between nuclei. Note that cells within 30 pixels of the image border were excluded from analysis in order to use the LCD feature.

Protrusions were determined by detecting regions in cytoplasm with intensity lower than 0.75 of average cytoplasm intensity based on the DHE/YAP channel to define the core of the cell body. Protrusion extent was defined as the proportion of the border of the cell core in contact with protrusive regions. Total protrusion area and features of the largest and second largest protrusions were also measured. Ruffliness was determined by the “edge” texture (see below) of DHE/YAP in the membrane region (kernel = 4 pixels) divided by cell roundness.

Texture features measure the distribution of pixel intensities in a cell or subcellular region. Haralick and Gabor features as well as SER (“Saddle/Edge/Ridge”, PerkinElmer) features were measured on DHE/YAP, actin, and DNA channels.

Mitotic cells were filtered from the data set using DAPI/Hoechst intensity, nucleus area, nuclear roundness, nuclear width, and DNA staining texture. Dead and poorly segmented cells were filtered based on ring region intensity, nucleus intensity, percent protrusion, cell area, nucleus area, and A_nuc_/A_cyto_. Segmentation was quality controlled for a selection of wells in each plate, particularly for wells with extreme phenotypes (e.g. large area, irregularly shaped nuclei) and/or low YAP, NF-κB, and DHE intensity.

*Reverse siRNA transfection*

Reverse transfection of siRNA was performed by first adding 10 ul Opti-MEM (Gibco) containing 40 nl Lipofectamine RNAiMax (Invitrogen) and 40 nl siRNA (20 mM) (or no siRNA) per well using a Multidrop dispenser (Thermo). After incubating plates at room temperature for 20 min, 30 ul of suspended cells were added to each well in transfection medium (4/3 concentrated growth medium, so that the final concentration of serum and additives was 1X). Cells were cultured in transfection medium for three days before fixation. For plating density experiments with cadherin knockdowns, cells were transfected for overnight in flasks using scaled up volumes of Lipofectamine RNAi Max and siRNA, then plated at varying concentrations in 384-well plates for two days before fixing and staining.

*mRNA quantification*

Total RNA was extracted using RNEasy Minikit (QIAGEN) according to manufacturer's protocol. 500 ng of purified RNA was converted to cDNA in a reaction containing 1mM dNTPs, 0.02mM DTT, 1U MMLV, 2U RNAse OUT (Invitrogen), and 0.3mM random primers (Promega), 90min at 37°C. Real time quantitative polymerase chain reaction (RT-qPCR) was performed using cDNA made from extracted RNA using SYBRGreen PCR Master Mix (Invitrogen) and fluorescence was measured using on a 7300 AB system (Applied Biosystems). ΔΔCt quantitation procedure was used from absolute measurements from triplicate samples. Intron-spanning primers for CDH1, CDH2, and beta-actin were designed using Primer-BLAST (NCBI) and oligonucleotides obtained from Sigma.

*Identification of significantly different morphological factors between NF-κB activation states*

For each cell line a random sample of 200 cells was drawn for each NF-κB state, i.e. cytoplasmic (log ratio < 0) or nuclear (log ratio > 0). Kolmogorov–Smirnov non-parametric tests were used to test the difference in 77 morphological features between the two samples for each cell line. Only features that were significantly different (*p* ≤ 0.05) in at least 10 cell lines were considered for further analysis.

*Bayesian modeling of NF-κB, YAP, and pJun*

A uniform random sample of 1000 cells (500 cells for each TF state, nuclear or cytoplasmic) was drawn from each cell line +/- TNFα. Each sample was composed of TF ratios and the 17 significant morphological features determined above (Fig. S2C). The ‘length of tallest protrusion’ feature was replaced with the more general 'total protrusions area' feature. Morphological features were log transformed and scaled between 0 and 1. Dependency models were generated using Biolearn network structure-learning tool, which searches for the network structure that maximize the model probability given the data (Sachs et al. 2005). For each sample, 1000 structure-learning searches were run using regression tree distribution and normal gamma scoring function. The resulting 1000 networks per cell line per condition were then averaged, and only edges that occurred in more than 60% (i.e. confidence ≥ 0.60) of the resulting networks are retained. In total, 60 models were generated for NF-κB, 10 models for YAP, and 20 models for pJun (+/- TNF).

*Prediction of average change in NF-κB using multivariate linear regression*

For each cell line and drug treatment combination (176 samples), the average fold change in the NF-κB ratios and morphological features relative to their controls (+/- TNFα) were calculated. All data were log transformed and scaled between 0 and 1. Morphological features whose fold change had a correlation greater than *R*^2^ = 0.4 with the fold change in NF-κB were selected to build a multiple linear regression model. The selected features were ruffliness, A_Nuc_ /A_Cyto_, and NF. 10-fold cross validation was performed to validate the model. Outliers were determined using the Matlab “regress” function.

**Supplementary Legends**

Figure S1. Characterization of cell lines. A) Comparison of features between morphological groups: L1 = cobblestone luminal, L2 = small luminal, B = Basal (mean +/- SD). * *p* < 0.05, ** *p* < 0.01. B) Representative images of cells stained to label N-cadherin (green), NF-κB (orange), and DNA (blue). Scale bar = 20 μm. C) Hierarchical clustering of replicate wells of 17 breast cancer cell lines (columns). Wells (14 replicates/cell line) were clustered by average Principal Component Analysis scores of cell shape features (uncentered correlation, average linkage) (rows). The 17 discrete clusters correspond to each of the cell lines. D-G) Well averages of cell area, nucleus area/cytoplasm area, neighbor fraction (NF), and Ruffliness for each cell line (+/- SD).

Figure S2. Heterogeneity in cell populations was used to identify relationships between morphological features and NF-κB localization. A) Distributions of nuclear/cytoplasmic NF-κB ratios in single cells, unstimulated (blue) and after TNFα stimulation (red = 1 h, green = 5 h). B) Distributions of first principal component scores of morphology of single cells using 77 shape features (unstimulated). Genetic subtypes are indicated by color and morphological clusters are indicated in parentheses. C) Matrix showing features whose values were significantly higher (yellow) or lower (cyan) in cells with high NF-κB ratio (nuclear NF-κB) than in cells with low NF-κB ratio (cytoplasmic NF-κB) in each line. D) Frequency of linear correlations between single cells morphology and NF-κB ratio in all cell lines for 77 morphological features (unstimulated).

Figure S3. YAP and Jun activation in a sub-set of 10 cell lines. A) Nuclear/cytoplasmic YAP ratios in each cell line with and without TNFα (mean +/- SD). B) Fold difference in nuclear phospho-Jun intensity (normalized to DAPI) in TNFα-stimulated (1 h) versus unstimulated cells. C) Example Bayesian network model for nuclear phospho-Jun showing connections between shape features, but no shape dependency for Jun activation with (red) or without (blue) TNFα. D) MCF10A cells expressing GFP-p65 were stimulated with TNFα (1 h) and stained for phospho-Jun. Nuclear/cytoplasmic GFP-p65 ratio plotted by nuclear phospho-Jun intensity normalized to Hoechst intensity (left) or nuclear/cytoplasmic phospho-Jun ratio (right). Each point represents a single cell. *n* = 594 GFP-positive cells.

Figure S4. Response of NF-κB to cytoskeleton modifying drugs. A) Nuclear/cytoplasmic NF-κB ratio for unstimulated and TNFα-stimulated (1 h and 6 h) in MCF10A cells. Y-27632 and blebbistatin doses are given in μM (5 h pre-treatment). Nocodazole doses are in μg/ml (2 h pre-treatment). Taxol doses are nM (2 h pretreatment). Noc+Y27 = 4 μM Y27 (5 h) plus 1 μg/ml nocodazole (2 h). N.S. indicates no significant difference from control. Unmarked bars are significantly different from control (*p* < 0.01). B) Difference between TNFα-stimulated and unstimulated NF-κB ratios. * significant difference from control (*p* < 0.01). C) Average NF-κB ratios plotted by first principle component (PC1) score of morphology features for drug treatments. D) MCF10A cells treated with nocodazole (0.1 μg/ml), Y-27632 (10 μM), blebbistatin (5 μM), or no drug (DMSO; control). Blue: Hoechst (DNA). Green: E-cadherin. Yellow: vimentin. Scale bar = 20 μm. E) Images from time-lapse imaging of cells transfected with GFP-p65 with or without drug treatment (m: minutes after addition of TNFα, 10 ng/ml). Scale bar = 20 μm. All contrast and brightness settings are the same across images.

Figure S5. Cell lines exhibit predicted dependencies between TF localization and plating density (NF). A-B) Frequencies of NF values in cells seeded at 500 (left) and 4000 (right) cells/well. B) NF-κB and YAP staining in high density well. Arrow: NF =1, high YAP. Arrowhead: NF = 1, low YAP ratio. C) NF-κB (left) and YAP (right) ratios (mean +/- SD) in cells with NF = 1 binned by local cell density (LCD). Correlation coefficients (log) (r) were determined for single cells from all wells in the plating density experiment (n = 5908). D and E) NF-κB (D) and YAP (E) nuclear/cytoplasmic ratios plotted by neighbor fraction. Single cells were binned by NF and the mean +/- SD for each bin are shown for each cell line. Regression lines were fitted and the slopes of these lines, which indicate the strength of the relationship, are shown in Figure 5. F) Linear correlations between TF ratio and NF for all single cells plated at varying densities by cell line. G) NF-κB ratio dependencies detected in Bayesian network models for wild type (solid red boxes) and RhoA knockdown (striped boxes) cells stimulated with TNFα (10 ng/ml, 1 h). RhoA-depleted MDA-MB-231 and SUM159 cells lost dependency on NF, whereas all cell lines kept or gained dependency on ruffliness.

Figure S6. Effects of substrate on cell morphology: Cell area (A) and nuclear area (B) of MCF10A (top) and MDA-MB-231 (bottom) cells on fibronectin-coated glass and polyacrylamide gels of different stiffness (kPa). Means +/- SD. ** denotes *p* < 0.01. * denotes *p* < = 0.5.

Figure S7. Characterization of the effects of N-cadherin knockdown in MCF10A cells. A) Representative images of mock transfected (top) and N-cadherin knockdown cells labeled for N-cadherin (green), actin (red) and DNA (blue). B) siRNA against N-cadherin specifically reduced the levels of target mRNA, but not E-cadherin mRNA, 72 hours after transfection. C) Average NF in wells with comparable numbers of cells (mean +/- SD). D) Nucleus area per NF bin (TNFα-stimulated cells). E) Nucleus area per NF bin (TNFα-stimulated cells). F) Nuclear/cytoplasmic NF-κB ratio per NF bin in unstimulated cells (mean +/- SD). G) Nuclear/cytoplasmic YAP ratio per NF bin in unstimulated cells. * difference from control *p* < 0.01. Unstimulated conditions: mock, n = 59410; E-cadherin k/d, n = 37471; N-cadherin k/d, n = 69413. Stimulated conditions: mock, n = 57334; E-cadherin k/d, n = 37660; N-cadherin k/d, n = 65515 (mean +/-SD).

**Supplementary Movies**

MCF10A cells transiently transfected with GFP-p65 were pre-treated with Y27632 (Supplementary Movies S3 and S4), nocodazole (Supplementary Movies S5 and S6), or no drug (Supplementary Movies S1 and S2; control) and stimulated with TNFα immediately prior to imaging. Images were taken every 5 min for 6 h.

**Supplementary Datasets**

Supplementary Dataset S1: Single cell data used to generate Bayesian network models for 19 cell lines +/- TNFα

Supplementary Dataset S2: Explanation of morphological features

Supplementary Dataset S3: Data used for multivariate linear regression (fold-change compared to control for each cell line)
