# Supplementary material for: Cell shape and the microenvironment regulate nuclear translocation of NF-κB in breast epithelial and tumor cells
Source: Mol Syst Biol. 2015 Mar 3;11(3):0790. doi: 10.15252/msb.20145644 (PMC4380925; doi:10.15252/msb.20145644)
Supplement: Supplementary file 16 [file msb0011-0790-sd16.pdf]

molecular systems biology

Cell Shape and the Microenvironment Regulate Nuclear Translocation of NF-κ B in Breast Epithelial and Tumor Cells

Julia E. Sero, Heba Zuhair Sailem, Rico Chandra Ardy, Hannah Almuttaqi, Tongli Zhang, Chris Bakal

Corresponding author: Julia Sero, Institute of Cancer Research

Review timeline:

Submission date:	01 August 2014
Editorial Decision:	08 September 2014
Revision received:	06 December 2014
Editorial Decision:	09 January 2015
Revision received:	16 January 2015
Accepted:	22 January 2015

Editor: Maria Polychronidou

Transaction Report:

(Note: With the exception of the correction of typographical or spelling errors that could be a source of ambiguity, letters and reports are not edited. The original formatting of letters and referee reports may not be reflected in this compilation.)

1st Editorial Decision 08 September 2014

Thank you again for submitting your work to Molecular Systems Biology. We have now heard back from two of the three referees whom we asked to evaluate your manuscript. Since their recommendations are rather similar, I prefer to make a decision now rather than further delaying the process. As you will see from the reports below, the reviewers acknowledge that the presented findings are potentially interesting. However, they list a number of issues and make suggestions for modifications, which we would ask you to carefully address in a revision of the manuscript. The referees' recommendations are clear in this regard.

On a more editorial level I would like to draw your attention to the following points:
- Our general journal policy is not to allow "data not shown". Therefore I would like to ask you to include the data related to the phenotypic changes induced by chemical inhibitors (described in p. 13). The data can be included as supplementary information.
- Please include a Conflict of Interest statement.
- Please provide a separate file containing the Supplementary Information (Supplementary Methods etc.).
- We would like to encourage you to provide the Source Data for the figures that show essential quantitative information. Additional information regarding source data is available in the "Author Guidelines" section in our website http://msb.embopress.org/authorguide#a3.4.
- When you resubmit your manuscript, please download our CHECKLIST (<http://msb.embopress.org/sites/default/files/additional-assets/EMBO%20Press%20Author%20Checklist%20-MSB.xlsx>) and include the completed form in your submission. For convenience reasons, the checklist is also attached below.

If you feel you can satisfactorily deal with these points and those listed by the referees, you may

© European Molecular Biology Organization

wish to submit a revised version of your manuscript. Please attach a covering letter giving details of the way in which you have handled each of the points raised by the referees. A revised manuscript will be once again subject to review and you probably understand that we can give you no guarantee at this stage that the eventual outcome will be favorable.

REFEREE REPORTS

Reviewer #2:

The question of whether variability in cell shape and population context contributes to variability in signaling responses to extracellular ligands is underexplored, in particular for pathways with no clear connection to mechanosensory inputs. Sero et al., tackled this question by using high content image analysis of fixed-cell images to quantitatively measure cell morphology and subcellular distribution of NFkB in untreated or TNF-treated single cells from a panel of 17 breast tumor cell lines and 2 breast non-tumor cell lines. Using Bayesian network models trained on their extensive datasets, they find that variability in specific cellular morphological features (e.g. 'ruffliness', contacts with neighbors) impacts NFkB subcellular distribution both before and after TNF treatment. They validate their approach by showing that it finds similar effects of cell shape on the distribution of YAP, a protein known to be affected by cellular morphology. By contrast, the authors found no impact of cell morphological features on the nuclear abundance of phospho-Jun. Finally, Sero et al. also test certain predictions from the models by chemical, biophysical, and genetic interventions and confirm by live-cell imaging that cell shape also affects the change in NFkB subcellular distribution upon TNF stimulation. The authors conclude that their data point to a role for cortical tension as regulated by the actomyosin cytoskeleton on NFkB activation and thus on gene expression.

The authors have collected an extensive dataset and applied it toward resolving questions that should be of broad interest in the systems biology community and also for researchers interested in signal transduction and in cancer biology. However, there are a few issues that need to be addressed to strengthen the manuscript and to add support to the conclusions stated by the authors.

1. Regarding the connections between NF-kB 'activity', gene expression and cell shape. In the opening paragraph of the results sections, the authors acknowledge that their measurement of "nuclear NF-kB translocation" "serves as a proxy for its activation". However, elsewhere they are not as careful in some of their statements (e.g. p. 25 "This work demonstrates the key role of shape and the microenvironment in regulating signaling transduction and gene expression"). To be able to make strong conclusions about gene expression or NF-kB activation, measurements of gene expression are warranted. For example, the authors could, in a hypothesis-driven way, use smFISH test how specific perturbations affect the correlation between cell morphology parameters and single-cell NF-kB induced transcript numbers. Alternatively, they could rely on RT-PCR measurements and show that after perturbations that lead to the most striking changes in cell morphology, the average NF-kB-induced transcript abundance changes in a way that is predicted by the average morphology changes. Finally, although this is a more detail oriented criticism, the word 'translocation' could be replaced by a more precise phrase here; 'subcellular distribution' is more appropriate because the data discussed are from fixed cells and therefore don't report on translocation per se. Throughout the manuscript, a careful choice of words could ensure that the links between data, interpretations, and conclusions are most credible to astute readers.

2. Regarding the determination of relationships between NF-kB and morphology in single cells, statistical analyses and Bayesian modeling. On p.45, the authors state "For each cell line a random sample of 200 cells was drawn for each NF-κB state, i.e. cytoplasmic (log ratio < 0) or nuclear (log ratio > 0). Kolmogorov-Smirnov non-parametric tests were used to test the difference in 77 morphological features between the two samples for each cell line...". Similarly, on p. 46 "A uniform random sample of 1000 cells (500 cells for each TF state, nuclear or cytoplasmic) was drawn from each cell line +/- TNFα." However, the distribution of ratio of Nuc/Cytoplasmic Intensity of p65 in un-stimulated cells varies dramatically among cell lines as they show in Fig. S2A. In some conditions, very few cells would score as cytoplasmic (e.g. SUM159, TNFα 1h), in others very few would be nuclear (e.g. T47D, unstimulated). In cases like these, the search for relationships therefore relies on the extreme outliers of the distributions. Would similar results be

arrived at if randomly sampling from the top and bottom quartile of each distribution? Or 0-10th and 90th-100th percentile intervals? Such an approach would be less likely to be biased by outliers and better take into account the spectrum of values exhibited under each scenario.

3. Regarding the analysis of the relationships between Jun phosphorylation and cell morphology. The authors found no significant associations between nuclear abundance of phosphorylated c-Jun in the presence or absence of TNF and any morphological features measured here. However, one important difference between how NF-kB and YAP data were analyzed compared with the phospho-Jun data is that the latter were normalized to DAPI intensity. The DAPI intensity is likely linked to nuclear area, nuclear roundness, etc... By normalizing to DAPI, the effects of cell morphology on phospho-Jun may be masked. This step in the analysis should better explained and validated or removed. Alternatively, are the effects on morphology for NF-kB subcellular distribution still detectable if the NF-kB data are normalized to DAPI intensity?

4. Finally, if phospho-Jun is indeed more uniquely affected by TNF receptor activation than NF-kB, the authors could take advantage of that finding by co-staining cells for RelA and phospho-Jun, and using the Jun signal to "normalize" by receptor activation, and derive an estimate of the impact of cell morphology on TNF-induced NF-kB subcellular distribution.

Other issues:

1. The authors should briefly justify the particular time points they chose to assess (1hr and 5hr post-TNF."

2. P. 9-10 (and Figure S2D). The authors assessed the linear correlations between NF-kB ratio and morphological features, but what are the Spearman correlation coefficients? They state the relationships are likely "complex and non-linear", but using Pearson correlation coefficients does not allow them to separate between the two, while assessing Spearman coefficients could identify any simple yet non-linear relationships.

3. P.14 and Fig. 4D: The data should be presented as a 2D scatter plot of "predicted vs. observed"; as it is currently presented, we cannot distinguish the two points (blue and red) for each case number and therefore the quality of the predictions cannot be ascertained. We can only see that the data spread only over a small dynamic range of NFkB ratio fold change. The authors also state "predictions were within the 95% confidence interval in all but seven cases."; do they mean instead that the observations were within the 95% CI of the predicted value?

4. P.17 and Fig. 5D and E; it would be more informative to have the real values of the slopes on the graph instead of the absolute values, otherwise the reader looses information about the direction of the effect. What are the confidence intervals on the estimated slopes? These should be displayed as error bars.

5. P.18: The authors state "These pathways may be altered in AU565 and HCC1954 cells which did not show NF-kB dependency on NF." To strengthen the model validation, the authors should assess the impact of knockdown of RhoA or treatment with Y27 in these cell lines - their conclusion suggests that there should be little impact, is that the case?

6. P.24: The authors write: "These results may help explain conflicting reports regading the role of the cytoskeleton in NF-kB regulation...". Please include citations.

7. P.25-26. The authors include some speculations in the discussion (for example on how shape-dependent regulation of NF-kB may have evolved), this should be more clearly represented as such ("we speculate that..."), to better distinguish from their conclusions that are specifically supported by their data.

8. While the work presented in this manuscript is clearly distinct, it would be important for the authors to discuss their findings in the context of prior reports from the Pelkmans group (in particular the last paragraph makes a point that has been specifically evaluated in Snijder et al., MSB 2012).

© European Molecular Biology Organization

9. P.27, 29: scrape wound assays were performed 3 days after seeding the cells, were all of the other assays timed similarly? On P.27, only the seeding density is indicated, not the post-seeding growth period.

10. A few suggested edits for the main text:
a. P.2: "Taken together these data cell shape tunes..." - missing a verb, perhaps instead "Taken together these data suggests that cell shape tunes..."

b. P.4: "... NF-kB has been observed to shuttle between the ..."; the use of the word 'shuttle' is a bit ambiguous here because it is sometimes used to describe the fact that NFkB molecules have been observed to 'shuttle' in and out of the nucleus constantly, even in unstimulated cells, even if its nuclear abundance does not change over short time scales. Perhaps instead "The nuclear to cytoplasmic ratio of NF-kB has been shown to exhibit dampened oscillations..."

c. P.4. To support the statement that "NF-kB activation and dynamics are heterogeneous on the single cell level even in isogenic populations", the authors cite Tay et al. 2010. However Nelson et al. 2004 (cited earlier in the same paragraph) were, I believe, the first to report and discuss this heterogeneity.

d. P.5: First use of the abbreviation "TF", it should be defined: "transcription factor (TF)".

e. P.8: "(LI) had significantly lower endogenous NFkB ratios..." or is what is meant here "un-stimulated NFkB ratios" or endogenous vs. exogenously expressed NFkB?

f. P.9: the B1 group" should be "the B group"; the L2 line HCC1954" should be " the L1 line HCC1954"; "the L1 line HCC70" should be "the L2 line HCC70"

g. P.14: In the first paragraph, the authors refer us to "(Methods)" but it should be to "(Supplementary experimental procedures)"

h. P.16: Instead of "... seeded at four different concentrations..." use "... seeded at four different densities..."

i. P.17: instead of "linear correlation... in all single MCF7 cells" use "linear correlation... in single MCF7 cells"

j. P.21: "The effects off cell shape" should be "The effects of cell shape"

11. Suggestions and corrections for figures:
a. Fig 1C: The color coding for Basal A and Basal B is difficult to distinguish; or completeness JIMT1 should be labeled as "unclassified"

b. Fig 1D: ANOVA statistics are presented in the text, but the figure only shows the distributions in Nuc/Cyto ratios as boxplots and thus should be labeled as such.

c. Fig 1E: T47D is mislabeled as L2; should be L1.

d. Fig 2C: some x-axis labels are missing

e. Fig 3D: it would be helpful to also note agreement/disagreement with NF-kB dependencies.

f. Fig 4C: Which statistical significance cut-off value was used?
g. Fig 4F, G: it would be helpful to also present the data as it is done in Fig 4I for the RhoA knockdown to show the quantitative relationship of Nc/Cyto with NF. Does the wound response alter the relationship that is observed in an unperturbed culture?

h. Fig 5D, E: show the real values (not absolute) of slopes along with their confidence intervals. If the CI excludes zero, this would strengthen the conclusion that NFis associated with NFkB distribution

12. Suggested changes in supplementary figures:
a. Fig S2A: include the morphology cluster next to each cell line, e.g. HCC70 (L2); CAMA1 (L2)

b. Fig S2D: there are unrecognizable characters on the axis of the graph

c. Fig S3C: the pJun model should include the pJun node, even if it is not connected to any node in the network(s)

d. Fig S4D: The list of cell lines used in the multivariate linear regression includes HeLa cells, although these are not mentioned in the methods and the rest of the experiments are focused on breast cancer cell lines? Why were they included here? How much do they influence the derivation of the model? Also, please indicate again the genetic subtype and morphology cluster information for each cell line (color code and cluster, L1, L2, B...).

e. Fig S4E the standard abbreviation for minutes should be "min", not "m" (meters).

Reviewer #3:

Sero and Sailem et al. investigate the influence of cell shape and environment on NF-kappaB signaling using high content image analysis and Bayesian network models in multiple breast cancer cell lines. The authors show that several physical factors, such as cell area, "ruffliness", and neighbor fraction affect p65/relA translocation intensity and dynamics, and suggest a strong mechanism and purpose for microenvironment-sensitive signaling.

Many single-cell signaling studies focus on heterogeneity in the cell population, and Sero et al. report on how this heterogeneity affects signaling and could have implications for response to tissue damage as well as epithelial-mesenchymal transition in breast cancer. This offers an unprecedented amount of cell shape and microenvironment parameters to explore and further informs the field on how to take into account shape parameters in future models and experiments. I feel this paper makes advances in both single cell image analysis techniques, in terms of the number of cells and parameters examined, and in understanding of how mechanisms of cell shape influence on the signaling behavior of NF-kappaB. I recommend it for publication in MSB, contingent on the following major and minor points being addressed:

Major Points:

1. The distinction between Basal A, Basal B, Luminal 1 and Luminal 2 clustering groups is unclear to the reader. I suggest an explanation of the different cell types, genetic subtypes, and naming consistency between Figures 1C-E to better understand how the cells are being classified, and how the clusters track across morphology and NF-kappaB activation. Further, the text discusses the ability of clustering to discriminate between Basal A and B but this is not evident from the Figure 1C, as the colors for Basal A and Basal B in this figure are also indistinguishable. Lastly, the beginning of the last paragraph on page 7/first paragraph page 8 says membership in a morphological cluster was not correlated with ER, PR, HER2 or TP53 status, but then goes on to say in the sixth sentence, that "morphological profiling was not only able to recapitulate genetic classfications,...". By genetic classifications, do the authors mean genetic subtype here (basal or luminal), because this could be confused with, for instance, HER2 genetic status? Please clarify the difference between luminal/basal classifications and genetic subtype and genetic status.

2. The experiments comparing NFkappaB activation to Jun activation support the conclusions that localization differences are not due to TNF detection, however, it would be further informative to show that the differences in p65 translocation intensity were not due solely to different TNF receptor levels across the cell lines. I suggest including this parameter for each cell line, either by IF or FACS.

3. The cell-scraping, substrate stiffness, RhoA k/d and ROCK, and N-cadherin studies are all well done and supportive to the idea that NFkappa B is sensitive to the microenvironment, however, they are only shown in the MCF10A cell line and in the substrate stiffness assay, the MCF10A and MDA-MB-231 cell lines. I recommend trying these in cell lines from each of the morphological

subtypes.

4. The authors have done a lot of work using genetic, chemical and physical perturbations to examine the influence of cell shape on signaling dynamics, but a powerful piece of data would be to take an epithelial breast cancer cell line and induce EMT under physiological conditions and then show how the alterations in cell shape track with in NFkappaB translocation. This can most likely be done by stimulating the cells with TGF-beta or overexpressing Snail. Conversely, the authors might try inducing mesenchymal to epithelial transition.

Minor Points:
1. There is a typo in the abstract, last sentence "Taken together, these data cell shape"

2. The introduction refers to "millions of cells" however, this is not clear from the manuscript. Please clarify.

3. Table 1 should be grouped to match the different genetic subtypes or morphology classifiers, as it is difficult for the readers to gather anything from the current alphabetized format.

4. Figure 1C and E - the colors used in the heatmaps are difficult to differentiate. Please use different colors. Also include a color key for 1C.

5. Figure 2A - repeatedly through the text and figures, please say the cell line used, and type of cell line so that readers do not have to refer back to Table 1.

6. Figures 2B and 3C are difficult to read. Please move labels to be more legible.

7. Figure 2C - are there labels missing on the x-axis? It also appears that some of the bars have different widths.

8. Figure 4A - please explain the order of the images. Additionally zoomed in images may be more informative at this scale.

9. Figure 4E - very difficult to read. Please use shaded error bars.

10. The 1995 Rosette and Karin paper referenced found Nocadazole increases nuclear p65. Please explain or suggest why you have conflicting results.

11. Figure 5G is said to have wave-like properties, however these are unclear and could be accounted for in the error bars.

12. In Figure 6D, the nuclei are difficult to pick out in the merged images. Please show the channels individually.

13. Please clarify how cellular cortical tension relates to substrate flexibility. Further, please describe which polyacrylamide gel is stiffer (readers may not understand this in terms of Young's modulus) and include a reference on their previous use.

14. The N-cadherin data in Figure 7 does not merit its own section, as it correlates with the earlier data on cell morphology. It should either be merged with earlier figures or moved to supplemental text.

15. The schematic in 7D should be made more informative. It should either be eliminated or more detailed in order to highlight the contribution of the paper to the field.

16. Supplemental Figure 2D - the axes labels are not legible

17. Supplemental Figure 3C - the legend refers to TNF for 0h and TNFa for 1h

18. Across the supplemental figures, NF-kappaB should be consistently referred to as NF-kappaB or

NF-κB, not NFkB (as in Supplementary Figures 2D and several in Figure S4).

1st Revision - authors' response 06 December 2014

Reviewer #2:

1. Regarding the connections between NF-kB 'activity', gene expression and cell shape. In the opening paragraph of the results sections, the authors acknowledge that their measurement of "nuclear NF-kB translocation" "serves as a proxy for its activation". However, elsewhere they are not as careful in some of their statements (e.g. p. 25 "This work demonstrates the key role of shape and the microenvironment in regulating signaling transduction and gene expression"). To be able to make strong conclusions about gene expression or NF-kB activation, measurements of gene expression are warranted. For example, the authors could, in a hypothesis-driven way, use smFISH test how specific perturbations affect the correlation between cell morphology parameters and single-cell NF-kB induced transcript numbers. Alternatively, they could rely on RT-PCR measurements and show that after perturbations that lead to the most striking changes in cell morphology, the average NF-kB-induced transcript abundance changes in a way that is predicted by the average morphology changes. Finally, although this is a more detail-oriented criticism, the word 'translocation' could be replaced by a more precise phrase here; 'subcellular distribution' is more appropriate because the data discussed are from fixed cells and therefore don't report on translocation per se. Throughout the manuscript, a careful choice of words could ensure that the links between data, interpretations, and conclusions are most credible to astute readers.

We have corrected the manuscript throughout to be more precise in terms of the description of the NF-kappaB localization phenotype that we have measured.

2. Regarding the determination of relationships between NF-kB and morphology in single cells, statistical analyses and Bayesian modeling. On p.45, the authors state "For each cell line a random sample of 200 cells was drawn for each NF-κB state, i.e. cytoplasmic (log ratio < 0) or nuclear (log ratio > 0). Kolmogorov-Smirnov non-parametric tests were used to test the difference in 77 morphological features between the two samples for each cell line...". Similarly, on p. 46 "A uniform random sample of 1000 cells (500 cells for each TF state, nuclear or cytoplasmic) was drawn from each cell line +/- TNFα." However, the distribution of ratio of Nuc/Cytoplasmic Intensity of p65 in un-stimulated cells varies dramatically among cell lines as they show in Fig. S2A. In some conditions, very few cells would score as cytoplasmic (e.g. SUM159, TNFα 1h), in others very few would be nuclear (e.g. T47D, unstimulated). In cases like these, the search for relationships therefore relies on the extreme outliers of the distributions. Would similar results be arrived at if randomly sampling from the top and bottom quartile of each distribution? Or 0-10th and 90th-100th percentile intervals? Such an approach would be less likely to be biased by outliers and better take into account the spectrum of values exhibited under each scenario.

The reviewer raises an interesting methodological point. We repeated the Bayesian network structure learning using the top and bottom quartile of each distribution. We found that the resulting networks were generally consistent with our previous results (see figure below). However, we observed that more morphological features were connected to NF-kB using the method suggested by the reviewer. We believe that this is because using only the top and bottom quartiles did not completely capture the dependency between morphological features. Consequently morphological features that affected NFkB indirectly were not depicted when only a limited range of the data was considered.

HCC1954 (-TNF) using bottom and top quartiles of NFkB ratios

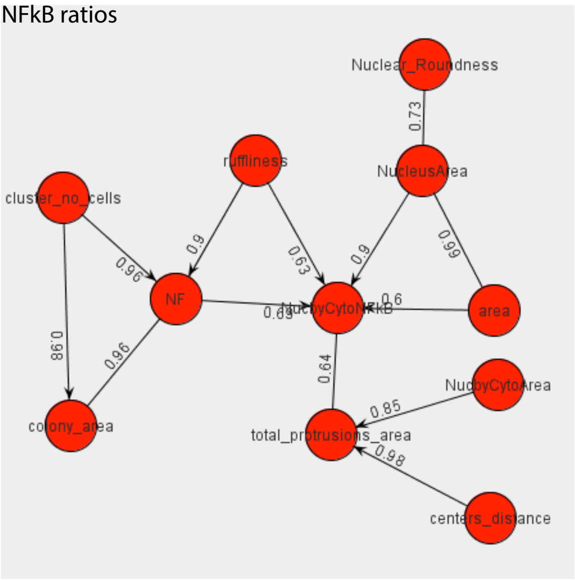

HCC1954 (TNF) using bottom and top quartiles of NFkB ratios

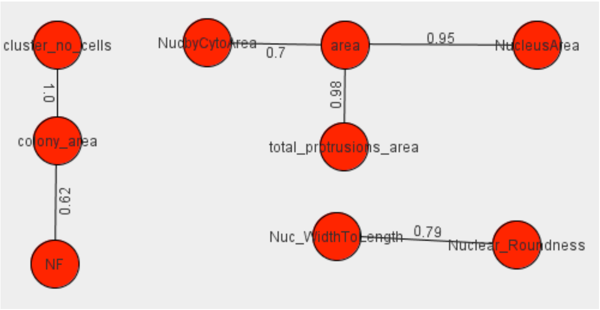

HCC1954 (-TNF(blue) and +TNF 1h (red)) using whole range of NFkB ratios

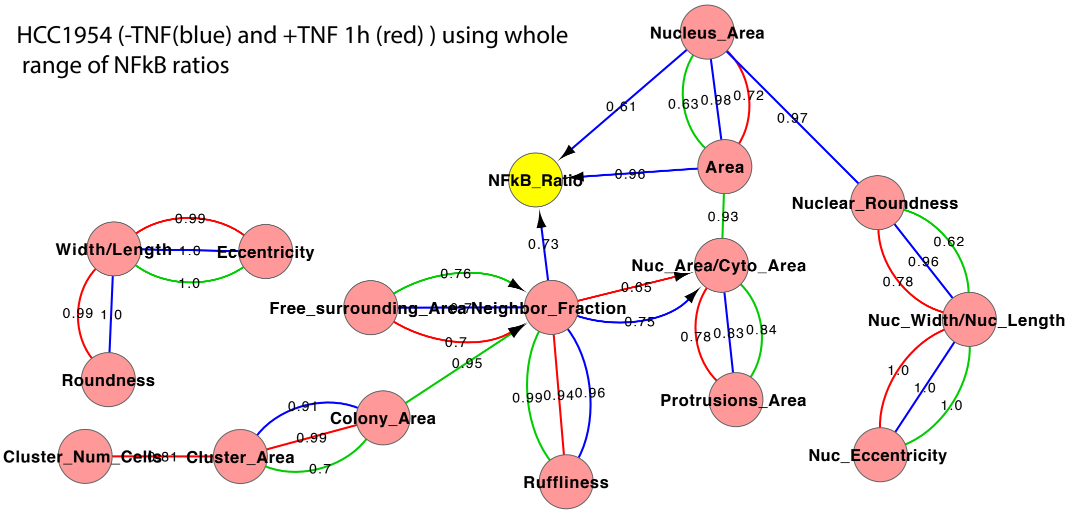

3. Regarding the analysis of the relationships between Jun phosphorylation and cell morphology. The authors found no significant associations between nuclear abundance of phosphorylated c-Jun in the presence or absence of TNF and any morphological features measured here. However, one important difference between how NF-kB and YAP data were analyzed compared with the phospho-Jun data is that the latter were normalized to DAPI intensity. The DAPI intensity is likely linked to nuclear area, nuclear roundness, etc... By normalizing to DAPI, the effects of cell morphology on phospho-Jun may be masked. This step in the analysis should better explained and validated or

© European Molecular Biology Organization

removed. Alternatively, are the effects on morphology for NF-kB subcellular distribution still detectable if the NF-kB data are normalized to DAPI intensity?

This is a good point. However, pJun was normalized to DAPI/Hoechst intensity in order to eliminate the effects of nucleus size (area and volume) on intensity. Specifically, we have observed that nuclei which are smaller in projected area (x,y) but taller in the z dimension tend to be brighter than nuclei which are more flattened. This effect seems to be a greater source of variation than DNA content, especially between cell lines. However, we repeated the Bayesian analysis as suggested, where we normalized nuclear pJun intensity to perinuclear pJun intensity. We still did not observe any dependency between pJun and morphological features. For example see below.

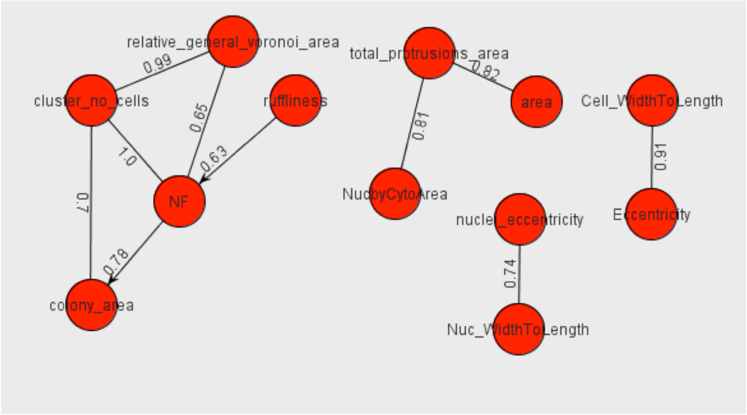

Network model for MDA-MB-157 cells using nuclear/cytoplasmic pJun intensity.

4. Finally, if phospho-Jun is indeed more uniquely affected by TNF receptor activation than NF-kB, the authors could take advantage of that finding by co-staining cells for RelA and phospho-Jun, and using the Jun signal to "normalize" by receptor activation, and derive an estimate of the impact of cell morphology on TNF-induced NF-kB subcellular distribution.

We thank the reviewer for this suggestion. Because both the NF-kB and pJun antibodies used for immunostaining, to address this point we transfected MCF10A cells with GFP-p65/RelA (which we showed in Fig. 4 to be responsive to TNFa and also sensitive to cell shape), stimulated with TNFa, fixed and stained for pJun, then quantified nuclear/cytoplasmic GFP-p65 together pJun. Plotting NF-kB ratio against either nuclear/cytoplasmic pJun or pJun/Hoechst intensity showed no significant correlation. We have not investigated heterogeneity in Jun activation in depth here as we feel it is outside the scope of the present work, so we hesitate to make a direct connection between receptor activation and Jun phosphorylation in these cells. We have included this data in Fig. S3D and have altered the text as follows:

"Furthermore, nuclear/cytoplasmic pJun and NF-κB ratios were not correlated in TNFα-stimulated MCF10A cells expressing GFP-p65/RelA (R^2 < 0.05, n = 594 GFP-positive cells) (Fig. S3D). (In comparison, the R^2 for NF-κB and YAP ratios was typically 0.3 for unstimulated MCF10A cells and 0.5 for TNFα-stimulated cells.)"

Other issues:

1. The authors should briefly justify the particular time points they chose to assess (1hr and 5hr post-TNF."

We have addressed this issue as follows:
"Cells were fixed at 1 and 5 h after addition of TNFα to capture the first peak and later steady state of NF-κB activation (see Fig. 4E)."

2. P. 9-10 (and Figure S2D). The authors assessed the linear correlations between NF-kB ratio and

morphological features, but what are the Spearman correlation coefficients? They state the relationships are likely "complex and non-linear", but using Pearson correlation coefficients does not allow them to separate between the two, while assessing Spearman coefficients could identify any simple yet non-linear relationships.

We estimated Spearman coefficients and found that correlation coefficients are still generally weak (although higher than Pearson correlations). Below is the distribution of Spearman correlation coefficients for all cell lines and all features. Bayesian networks are again advantageous here, as they allow us to estimate relationships based on conditional probabilities and detect complex relationships between variables.

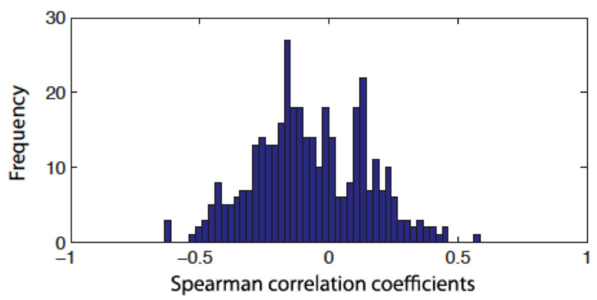

Distribution of Spearman coefficients (compare to distribution of Pearson coefficients in Fig. S2D).

3. P.14 and Fig. 4D: The data should be presented as a 2D scatter plot of "predicted vs. observed"; as it is currently presented, we cannot distinguish the two points (blue and red) for each case number and therefore the quality of the predictions cannot be ascertained. We can only see that the data spread only over a small dynamic range of NFkB ratio fold change. The authors also state "predictions were within the 95% confidence interval in all but seven cases."; do they mean instead that the observations were within the 95% CI of the predicted value?

We have replaced Fig. 4D by a 2D scatter plot of "predicted vs. error" as suggested by the reviewer to make the figure more clear to the readers.

We also changed the text to “only seven cases were not within the 95% confidence interval of the predicted value”.

4. P.17 and Fig. 5D and E; it would be more informative to have the real values of the slopes on the graph instead of the absolute values, otherwise the reader looses information about the direction of the effect. What are the confidence intervals on the estimated slopes? These should be displayed as error bars.

We have corrected the figure as suggested and included error bars representing the SD of the measured slopes for each condition.

5. P.18: The authors state "These pathways may be altered in AU565 and HCC1954 cells which did not show NF-kB dependency on NF." To strengthen the model validation, the authors should assess the impact of knockdown of RhoA or treatment with Y27 in these cell lines - their conclusion suggests that there should be little impact, is that the case?

This is an interesting point. NF-kB was not dependent on NF in AU565 and HCC1954 cells under normal or ROCK-inhibited conditions, but we to address this question we tested the effects of RhoA knockdown in four cancer cell lines. Two lines that did show this dependency (MDA-MB-231 and SUM159) lost that relationship after knockdown of RhoA. The text has been altered to include data

on four other RhoA-depleted cell lines. The manuscript has been altered on page 18 as follows:

"RhoA knockdown also eliminated the NF dependency observed in wild type MDA-MB-231 (Basal B, B) and SUM159 (Basal B, L/B) cells stimulated with TNFα (1 h) (Fig. S5G). However, NF-κB ratio dependency on ruffliness was observed in these lines (confidence > 0.9), as well as in RhoA-depleted AU565 (Luminal, L2) and T47D (Luminal, L1) cells, in which NF-κB was not normally connected to NF. Taken together, these data suggest that the RhoA-ROCK pathway is involved in mediating the negative regulation of NF-κB by cell-cell contact, and that this link may be broken in some cancer cells."

The text on page 15 has been altered as follows to include specific data on drug treated cells and to better explain the use of the multivariate linear regression model.

"In general, NF-κB ratios increased in cancer cell lines treated with ROCK inhibitors and decreased in cells treated with nocodazole, but different lines showed different sensitivities to drugs (See Supplementary Data). To determine whether changes in cell shape could explain the changes in NF-κB localization, we used multivariate linear regression with 10-fold cross validation to predict the fold change in NF-κB ratio from the fold change in morphological features."

6. P.24: The authors write: "These results may help explain conflicting reports regading the role of the cytoskeleton in NF-kB regulation...". Please include citations.

Citations have been included.

7. P.25-26. The authors include some speculations in the discussion (for example on how shape-dependent regulation of NF-kB may have evolved), this should be more clearly represented as such ("we speculate that..."), to better distinguish from their conclusions that are specifically supported by their data.

The text has been altered as suggested.

8. While the work presented in this manuscript is clearly distinct, it would be important for the authors to discuss their findings in the context of prior reports from the Pelkmans group (in particular the last paragraph makes a point that has been specifically evaluated in Snijder et al., MSB 2012).

The text has been altered as followed to address this point directly:

"Finally, these studies illustrate the utility of Bayesian network modeling for uncovering complex relationships between cell form and function, and they highlight the importance of context in determining cell behavior. Cellular context was shown to be an important source of variation in virus infectivity and endocytosis, and accounting for such differences in cellular states could explain much of the heterogeneity observed within cellular populations (Snijder et al, 2009). These findings indicate that cell shape and context are important determinants of transcription factor regulation and cells' response to chemical signals. Thus, care should be taken to consider factors such as density when interpreting data from experiments where conditions may vary, including RNAi screens, drug screens, and comparisons between cell lines."

9. P.27, 29: scrape wound assays were performed 3 days after seeding the cells, were all of the other assays timed similarly? On P.27, only the seeding density is indicated, not the post-seeding growth period.

All experiments were performed on the third day in culture unless otherwise specified. The text has been corrected to include this.

10. A few suggested edits for the main text:
a. P.2: "Taken together these data cell shape tunes..." - missing a verb, perhaps instead "Taken together these data suggests that cell shape tunes..."

Corrected.

© European Molecular Biology Organization

b. P.4: "... NF-kB has been observed to shuttle between the ..."; the use of the word 'shuttle' is a bit ambiguous here because it is sometimes used to describe the fact that NFkB molecules have been observed to 'shuttle' in and out of the nucleus constantly, even in unstimulated cells, even if its nuclear abundance does not change over short time scales. Perhaps instead "The nuclear to cytoplasmic ratio of NF-kB has been shown to exhibit dampened oscillations..."

Text changed as suggested.

c. P.4. To support the statement that "NF-kB activation and dynamics are heterogeneous on the single cell level even in isogenic populations", the authors cite Tay et al. 2010. However Nelson et al. 2004 (cited earlier in the same paragraph) were, I believe, the first to report and discuss this heterogeneity.

Citations were switched – corrected.

d. P.5: First use of the abbreviation "TF", it should be defined: "transcription factor (TF)".

Corrected.

e. P.8: "(LI) had significantly lower endogenous NFkB ratios..." or is what is meant here "un-stimulated NFkB ratios" or endogenous vs. exogenously expressed NFkB?

Corrected to "unstimulated".

f. P.9: the B1 group" should be "the B group"; the L2 line HCC1954" should be " the L1 line HCC1954"; "the L1 line HCC70" should be "the L2 line HCC70"

Corrected.

g. P.14: In the first paragraph, the authors refer us to "(Methods)" but it should be to "(Supplementary experimental procedures)"

Corrected.

h. P.16: Instead of "... seeded at four different concentrations..." use "... seeded at four different densities..."

Corrected.

i. P.17: instead of "linear correlation... in all single MCF7 cells" use "linear correlation... in single MCF7 cells"

Corrected.

j. P.21: "The effects off cell shape" should be "The effects of cell shape"

Corrected.

11. Suggestions and corrections for figures:
a. Fig 1C: The color coding for Basal A and Basal B is difficult to distinguish; or completeness JIMT1 should be labeled as "unclassified"

Changed color of Basal A cell lines to green and labeled JIMT1 as "unclassified" in all figures.

b. Fig 1D: ANOVA statistics are presented in the text, but the figure only shows the distributions in Nuc/Cyto ratios as boxplots and thus should be labeled as such.

Corrected.

© European Molecular Biology Organization

c. Fig 1E: T47D is mislabeled as L2; should be L1.

Corrected.

d. Fig 2C: some x-axis labels are missing

Corrected.

e. Fig 3D: it would be helpful to also note agreement/disagreement with NF-kB dependencies.

We have noted in the text that the features most commonly connected to both NF-kB and YAP were ruffliness and neighbor fraction, as well as nuclear morphology.

f. Fig 4C: Which statistical significance cut-off value was used?

The cut-off was $p < 0.01$; this has been included in the figure legend.

g. Fig 5F, G: it would be helpful to also present the data as it is done in Fig 4I for the RhoA knockdown to show the quantitative relationship of Nc/Cyto with NF. Does the wound response alter the relationship that is observed in an unperturbed culture?

The relationship between NF and NF-kappaB ratio is similar to that shown in Fig, 4I, but in the interest of showing multiple time-points, we chose to present the data as edge vs non-edge cells. However, for clarity we have altered the figure to show that "edge" cells have NF 0.3-0.8 and "non-edge" cells have NF = 1.

h. Fig 5D, E: show the real values (not absolute) of slopes along with their confidence intervals. If the CI excludes zero, this would strengthen the conclusion that NFis associated with NFkB distribution

Real (negative) slope values are shown with error bars depicting standard deviation of slopes for each condition.

12. Suggested changes in supplementary figures:
a. Fig S2A: include the morphology cluster next to each cell line, e.g. HCC70 (L2); CAMA1 (L2)

Corrected as per suggestion.

b. Fig S2D: there are unrecognizable characters on the axis of the graph

Corrected.

c. Fig S3C: the pJun model should include the pJun node, even if it is not connected to any node in the network(s)

Corrected.

d. Fig S4D: The list of cell lines used in the multivariate linear regression includes HeLa cells, although these are not mentioned in the methods and the rest of the experiments are focused on breast cancer cell lines? Why were they included here? How much do they influence the derivation of the model? Also, please indicate again the genetic subtype and morphology cluster information for each cell line (color code and cluster, L1, L2, B...).

In parallel with these studies, we also characterized the Hela cell line because it is well-studied cancer model. Running the multilinear regression model without including Hela cell line gave similar results. R^2=0.33, p-value= 2.8e-13, and estimate of error= 0.038. We added a comment in the text to highlight the fact that NF-kB behaves similarly in Hela cells. We also added this line to the experimental methods.

e. Fig S4E the standard abbreviation for minutes should be "min", not "m" (meters).

Corrected.

Reviewer #3:

Major Points:

1. The distinction between Basal A, Basal B, Luminal 1 and Luminal 2 clustering groups is unclear to the reader. I suggest an explanation of the different cell types, genetic subtypes, and naming consistency between Figures 1C-E to better understand how the cells are being classified, and how the clusters track across morphology and NF-kappaB activation. Further, the text discusses the ability of clustering to discriminate between Basal A and B but this is not evident from the Figure 1C, as the colors for Basal A and Basal B in this figure are also indistinguishable. Lastly, the beginning of the last paragraph on page 7/first paragraph page 8 says membership in a morphological cluster was not correlated with ER, PR, HER2 or TP53 status, but then goes on to say in the sixth sentence, that "morphological profiling was not only able to recapitulate genetic classfications,...". By genetic classifications, do the authors mean genetic subtype here (basal or luminal), because this could be confused with, for instance, HER2 genetic status? Please clarify the difference between luminal/basal classifications and genetic subtype and genetic status.

The figure has been altered to make classifications more clear, and the text has been corrected to clarify that we mean genetic subtype (basal/luminal) determined by gene expression profile.

2. The experiments comparing NFkappaB activation to Jun activation support the conclusions that localization differences are not due to TNF detection, however, it would be further informative to show that the differences in p65 translocation intensity were not due solely to different TNF receptor levels across the cell lines. I suggest including this parameter for each cell line, either by IF or FACS.

This is a good point that we have also considered. Immunolabeling experiments with anti-TNFR1 were inconclusive, however, so we decided to examine a gene expression dataset generated from the same cell lines we used in our experiments (Grigoriadis et al, BMC Genomics 2012). Using mRNA expression data for 18 of the cell lines (no data was available for AU565), we looked for correlations (Spearman coefficients) between NF-kB ratios in each condition and over 28,000 genes. Specifically, we did not find any significant correlations between NF-kB and the expression of various TNFalpha-related genes or with components of the canonical NF-kB signaling pathway (Table SI).

3. The cell-scraping, substrate stiffness, RhoA k/d and ROCK, and N-cadherin studies are all well done and supportive to the idea that NFkappa B is sensitive to the microenvironment, however, they are only shown in the MCF10A cell line and in the substrate stiffness assay, the MCF10A and MDA-MB-231 cell lines. I recommend trying these in cell lines from each of the morphological subtypes.

We tried to perform the soft substrate experiment in luminal cell lines (e.g T47D), but found that these cells would not adhere and spread on the PA gels. Instead, they formed spheroids that detached and floated in the medium even on 35 kPa substrates.

4. The authors have done a lot of work using genetic, chemical and physical perturbations to examine the influence of cell shape on signaling dynamics, but a powerful piece of data would be to take an epithelial breast cancer cell line and induce EMT under physiological conditions and then show how the alterations in cell shape track with in NFkappaB translocation. This can most likely be done by stimulating the cells with TGF-beta or overexpressing Snail. Conversely, the authors might try inducing mesenchymal to epithelial transition.

We agree that this would be a very interesting piece of data! Unfortunately, due to technical issues we have been unable to perform this experiment more than once in the time frame of resubmission, so we have not included the data in the manuscript. However, we did compare MCF10A cells treated with TGF-beta (10 ng/ml for 6 days, including one passage, followed by 3 days culture in 384-well plates at varying densities) to untreated control cells grown in parallel, and found that there

was not a significant difference in NF-kB ratio between groups following TNFa stimulation when cells were binned by Neighbor Fraction (see below).

This is consistent with our model, in which the effect of EMT on the NF-kB response is due to the accompanying shape changes, rather than global alterations in gene expression. Indeed, our data suggest that we can mimic an EMT-like NF-kB phenotype (i.e. increased nuclear localization in response to TNFa) simply by altering cell shape through cytoskeletal disruption and changing the microenvironment.

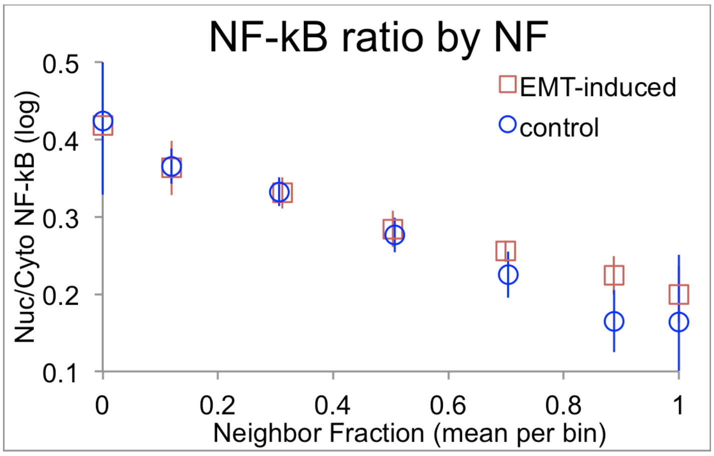

TGFβ-treated and control cells stimulated with TNFα (10 ng/ml, 1 h) binned by NF (means per bin +/- SD; n = 12320 control and 7814 EMT-induced cells).

Minor Points:
1. There is a typo in the abstract, last sentence "Taken together, these data cell shape"

Corrected.

2. The introduction refers to "millions of cells" however, this is not clear from the manuscript. Please clarify.

The text has been changed to:
"We used high content analysis (HCA) to quantitatively measure cell morphology and transcription factor (TF) localization in hundreds of thousands of single cells per experiment in cell lines derived from human breast tumor and non-tumor tissues."

(The total number of cells analyzed for this paper is well over a million.)

3. Table 1 should be grouped to match the different genetic subtypes or morphology classifiers, as it is difficult for the readers to gather anything from the current alphabetized format.

Cell lines are grouped by genetic subtype.

4. Figure 1C and E - the colors used in the heatmaps are difficult to differentiate. Please use different colors. Also include a color key for 1C.

A color key has been included in Fig. 1C. We used blue-yellow heatmaps instead of red-green to accommodate for color blind readers.

5. Figure 2A - repeatedly through the text and figures, please say the cell line used, and type of cell line so that readers do not have to refer back to Table 1.

This information has been included throughout the text.

6. Figures 2B and 3C are difficult to read. Please move labels to be more legible.

Corrected.

7. Figure 2C - are there labels missing on the x-axis? It also appears that some of the bars have different widths.

Corrected.

8. Figure 4A - please explain the order of the images. Additionally zoomed in images may be more informative at this scale.

The order of images has been explained in the figure legend (by PC1 value as in Fig. 4B) in the figure legend and more zoomed images have been used.

9. Figure 4E - very difficult to read. Please use shaded error bars.

We have altered the error bars and colors to improve readability.

10. The 1995 Rosette and Karin paper referenced found Nocadazole increases nuclear p65. Please explain or suggest why you have conflicting results.

The text has been altered to read:

"We found that nocodazole generally increased NF-κB nuclear localization in unstimulated cells, similar to Rosette and Karin (1995); however, this could be due to the induction of cellular stress responses to drug treatment."

11. Figure 5G is said to have wave-like properties, however these are unclear and could be accounted for in the error bars.

We have removed this sentence.

12. In Figure 6D, the nuclei are difficult to pick out in the merged images. Please show the channels individually.

We are not sure to what the reviewer is referring to, as there is no Fig. 6D.

13. Please clarify how cellular cortical tension relates to substrate flexibility. Further, please describe which polyacrylamide gel is stiffer (readers may not understand this in terms of Young's modulus) and include a reference on their previous use.

We have included references to PA gels, clarified the relative stiffness of the gels used, and included references to the relationship between matrix stiffness, cortical tension and cell shape in the Results section. The text has been altered as follows:

"Based on these findings, we hypothesized that cortical F-actin could couple NF-κB activation to protrusion and spreading. Specifically, nuclear translocation could be enhanced in cells that are under low cortical tension, such as protrusive cells or cells at the edge of a colony, and suppressed in round or non-protrusive cells with high cortical tension (Maddox and Burridge, 2003; Thoumine et al., 1999). To test this hypothesis, we cultured MCF10A and MDA-MB-231 cells on fibronectin-coated glass or flexible polyacrylamide (PA) gels with Young's moduli (a measure of stiffness) of 35 and 16 kPa (Tse and Engler, 2010) and measured cell shape and NF-κB localization in cells stimulated with TNFα. PA gels have long been used to fabricate substrates with more physiologically relevant elasticity than glass (Pelham and Wang, 1997). Substrate stiffness can control differentiation, motility, and cytoskeleton organization as cells sense and respond to matrix compliance (reviewed by Discher et al Science 2005). Moreover, cells can tune their internal stiffness to match that of the extracellular matrix by controlling F-actin crosslinking and contraction (Solon et al., 2007)."

14. The N-cadherin data in Figure 7 does not merit its own section, as it correlates with the earlier data on cell morphology. It should either be merged with earlier figures or moved to supplemental text.

We respectfully disagree, as this figure demonstrates that by changing expression of an EMT marker that correlates with our morphological clusters as well as NF-kB ratios, we see predictable changes

© European Molecular Biology Organization

both in shape and NF-kB response to TNFa.

15. The schematic in 7D should be made more informative. It should either be eliminated or more detailed in order to highlight the contribution of the paper to the field.

We have removed the schematic and included a "visual blurb" which we feel is more informative.

16. Supplemental Figure 2D - the axes labels are not legible

Corrected.

17. Supplemental Figure 3C - the legend refers to TNF for 0h and TNFa for 1h

Corrected.

18. Across the supplemental figures, NF-kappaB should be consistently referred to as NF-kappaB or NF-κB, not NFkB (as in Supplementary Figures 2D and several in Figure S4).

Corrected.

2nd Editorial Decision 09 January 2015

Thank you again for submitting your work to Molecular Systems Biology. We have now heard back from the two referees who were asked to evaluate your manuscript. As you will see, the referees are now satisfied with the modifications made. However, they list a series of minor points that we would ask you to address in a revision of the manuscript. Moreover, reviewer #2 refers to the need to include some further explanations regarding their previously raised point, on the normalization of the Jun and NF-kB signals.

As a matter of course, please make sure that you have correctly followed the instructions for authors as given on the submission website.

Thank you for submitting this paper to Molecular Systems Biology.

REFEREE REPORTS

Reviewer #2:

We were pleased to read the revised manuscript from Sero et al. and noted several improvements, in particular the inclusion of a few pieces of new data and clarifications to the figures.

In our opinion, one potential issue remains, relating to our original point #3 about the different normalizations the Jun vs. NF-kB immunofluorescence signals. In their response, the authors state that "pJun was normalized to DAPI/Hoechst intensity in order to eliminate the effects of nucleus size (area and volume) on intensity." Isn't the effect of nucleus size also of concern for the intensity of NF-kB and YAP? Similarly cytoplasmic area should impact cytoplasmic intensity, an important consideration because nuclear to cytoplasmic ratio of NF-kB is estimated as log(Nuclear intensity/Ring region intensity).

Specifically, one might expect ring region intensity to be strongly affected by cytoplasmic area (particularly in widefield imaging, a flattened cell occupying a large area could have much lower ring region intensity than a cuboidal cell of the same volume). Therefore the variation in NF-kB ratio as defined could directly reflect variation in cell shape, instead of variation in actual partitioning of NF-kB (log(total nuclear/total cytoplasmic NF-kB)). Indeed the highest frequency of NF-kB dependency is on cell area (Fig 2C-D). While the various perturbations performed (with inhibitors, knockdowns) have a real impact on the measured log(Nuclear intensity/Ring region intensity), it seems that some of it could be explained via their effect on cell shape. Possible ways to circumvent this issue include: 1) using confocal imaging to capture the intensity at a single focal

plane through the nucleus and cytoplasm (fluorescence intensity would thus measure "concentration" in a set volume; the authors note that they are using a microscope with confocal capabilities, but it's unclear if the images were captured in widefield or confocal mode) or 2) using instead the log(integrated nuclear intensity/integrated cytoplasmic intensity).

Although we are concerned that the effects of cell shape on NF-kB subcellular distribution that the authors have observed may be in part due to their particular measure of nuclear to cytoplasmic ratio of NF-kB, we recognize that the effect of cell features on the measured dynamics of NF-kB translocation (timing of initial peak and oscillation frequency) are much less likely to be a simple artifact of the measurement approach. In addition, the fact that the nuclear to cytoplasmic ratios of NF-kB and YAP are only loosely correlated also provides reassurance (the data on ratio for pJun perhaps less so because the cytoplasmic staining seems very close to background and therefore may be more subject to noise). Nevertheless, a clearer justification for the authors' choice of metric (log(Nuclear intensity/Ring region intensity)) and their choice to normalize only the pJun signal is warranted.

Minor issues:

• Regarding the use of the phrase "NF-kB translocation" - in our opinion for clarity this should be reserved to refer to a *change* in localization over time (for example when describing fold-change in NF-kB nuclear to cytoplasmic ratio after TNF treatment).

• Figure S3 has not been updated and therefore is missing the new panel D.

• P.5 - "recent studies appear to suggest these oscillations may be cell-type specific" - please include references.

• p.8 - "Replicate wells also clustered together, showing good reproducibility of feature measurements (not shown)." - this clustering result should be shown in the supplementary materials.

Reviewer #3:

Based on the issues raised in the previous review I am satisfied with the changes that were made to make the manuscript more reader friendly and the figures clearer. The descriptions of the PA experiments, in particular, is much more accessible. In addition, I am satisfied that the authors attempted to do some of the experiments suggested in the major points and appreciate that some of them were difficult to either accomplish or finish by the deadline. I have one remaining issue that must be addressed before publication, and contingent upon this change, I feel the paper is suitable for publication.

Major Point:

The "Highlights" portion of the text claims "EMT enhances NF-κB activation through changes in cell shape". However, this is never addressed in the main text or substantiated in the data. I believe the N-cadherin data demonstrates there is a clear link between cell shape and NF-κB localization, however, there is no direct link with EMT. I understand there is a correlated link, however, the statement is too strong for the data presented, even taking into account the data presented to the reviewers in the resubmission (Major Point 4). The paper makes a solid case that cell shape can drive NF-κB localization, and this alone is a significant finding. If the authors still would like to include this statement as is, experiments need to be included that would provide evidence of a direct link from EMT to NF-κB localization changes.

Minor Points:

1. Throughout the text and figures, TNFα is referred to as TNFa. This should be corrected throughout.

2. The following typos should be corrected:

a. Pg 15. 3rd line from the end, "To further validate... expected alter to..." should be "expected to alter"

b. Pg 28. Line 10. "Further high... studies the..." should be "studies that"

3. Figure 7A. It would be helpful to provide images of the individual channels to ascertain the changes in NF-κB localization. This could either be included in this figure or in the supplementary materials.

4. Figure 7B right. While the arrow is noted in the text it would be useful to also add it in the figure legend.

2nd Revision - authors' response 16 January 2015

Response to Reviewer 2:

Although they do not dispute the findings or interpretation of the results, Reviewer 2 raises some issues about the method of normalization for pJun intensity and NF-kappaB. First of all, the nucleus/ring region intensity measurement was used for NF-kappaB (and YAP) because this method essentially "normalizes" for differences in total intensity (protein level) from cell to cell. Second, the ring region area was used instead of the total cytoplasm intensity precisely for the reasons the reviewer pointed out – because differences in cell area would have an inordinate effect on average intensity due to the thickness of a spread versus round cell. The ring region was defined as a ring about 3 microns wide around the nucleus. This small region will for the most part be of comparable thickness in the z direction to the nuclear region, whether cells were spread or round. These studies were performed using an Opera automated spinning disk confocal microscope (with no widefield fluorescence capabilities) and NF-kappaB/YAP/pJun staining was performed using a 20X objective lenses with NA = 0.45, which means that the z-resolution in the focal plane was on the order of several microns. While the proportion of protein located in the ring region to the total cytosolic protein may be slightly less for very flat cells compared with very round cells, this seemed to be balanced out by differences in nuclear thickness in z. Therefore, we feel that the ring region intensity is reasonably representative of cytoplasmic total for the purposes of these studies, and further normalization for protein content is not necessary. We also experimented with using integrated intensities, but found that these measurements were actually noisier than regional averages, due particularly to the facts that depending on the shape of the cell, 1) the ring region areas could be significantly different from one another (not always ring-shaped if the nucleus was proximal to the edge of the cell) and 2) the entire cytoplasm regions were often not contained in the ~3-4 um focal "plane" corresponding to approximate average center of nuclei for a given cell line. Integrated cytoplasmic intensity could be better approximated using z-stacks (and deconvolution), but this was outside the scope of the present study.
In terms of pJun intensity measurements, we decided to normalize to DAPI/Hoechst intensity precisely for the reason that the reviewer suggested, namely that the cytoplasmic intensities may have been too low to give meaningful "self-normalized" ratios. We also experimented with using integrated intensities for these measurements, but decided to normalize to the DNA label because nuclear intensities in differently-shaped were strongly correlated with nuclear area. We did not find any correlation between cell shape features and pJun staining using either these calculations or nuclear/cytoplasmic intensity. The ideal normalization control would have been total Jun protein, but unfortunately this was not possible for these experiments. If we were to focus on Jun activation, this would be a parameter to optimize fully; however, we feel that the pJun normalizations we reported, and those suggested by the reviewers, were sufficient for the purposes of this study.

We have changed the Expanded Experimental Procedures text to read:

"Nuclear pJun intensity was normalized to nuclear DAPI/Hoechst intensity because cytoplasmic levels of phosphorylated Jun were very low. However, the same network modeling results were obtained using the log(Nuclear/Ring region intensity) measurement for pJun."

Minor points:

1) We have altered “nuclear translocation” to “nuclear localization” in the Results section where we are not referring to fold-change measurements.

2) A revised Fig. S3 which includes the missing panel is submitted.

3) References added on page 5.

4) Well-by-well clustering added to Supplemental Figures (Fig. S1C).

Response to Reviewer 3:

Major Point

We have altered the “Highlights” to remove the bullet point about EMT as this has not been explicitly demonstrated here.

Minor points:

1. In the .docx files we submitted TNFα is labelled as such (was there a .pdf conversion issue?), and instances of “TNFa” has been corrected to “TNFα” in the figures.

2. The noted corrections have been made.

3-4. Single channel images in grayscale we added in Fig. 7 and the figure legend has been corrected.

© European Molecular Biology Organization
